# Aerobic, resistance, and mind-body exercise are equivalent to mitigate symptoms of depression in older adults: A systematic review and network meta-analysis of randomised controlled trials

**DOI:** 10.12688/f1000research.27123.1

**Published:** 2020-11-13

**Authors:** Kyle J. Miller, Pinyadapat Areerob, Declan Hennessy, Daniela C. Gonçalves-Bradley, Christopher Mesagno, Fergal Grace

**Affiliations:** 1 School of Health and Life Sciences, Federation University, Ballarat, Victoria, 3350, Australia; 2 Nuffield Department of Population Health, University of Oxford, Oxford, UK

**Keywords:** Older adults, elderly, seniors, exercise, physical activity, depression, randomised controlled trial, RCT

## Abstract

**Background:** Exercise has been identified as an allied health strategy that can support the management of depression in older adults, yet the relative effectiveness for different exercise modalities is unknown. To meet this gap in knowledge, we present a systematic review and network meta-analysis of randomised controlled trials (RCTs) to examine the head-to-head effectiveness of aerobic, resistance, and mind-body exercise to mitigate depressive symptoms in adults aged ≥ 65 years.

**Methods:** A PRISMA-NMA compliant review was undertaken on RCTs from inception to September 12 ^th^, 2019. PubMed, Web of Science, CINAHL, Health Source: Nursing/Academic Edition, PsycARTICLES, PsycINFO, and SPORTDiscus were systematically searched for eligible RCTs enrolling adults with a mean age ≥ 65 years, comparing one or more exercise intervention arms, and which used valid measures of depressive symptomology. Comparative effectiveness was evaluated using network meta-analysis to combine direct and indirect evidence, controlling for inherent variation in trial control groups.

**Results:** The systematic review included 81 RCTs, with 69 meeting eligibility for the network meta-analysis ( *n* = 5,379 participants). Pooled analysis found each exercise type to be effective compared with controls (Hedges’ *g* = -0.27 to -0.51). Relative head-to-head comparisons were statistically comparable between exercise types: resistance versus aerobic (Hedges’ *g* = -0.06, *PrI* = -0.91, 0.79), mind-body versus aerobic (Hedges’ *g* = -0.12, *PrI* = -0.95, 0.72), mind-body versus resistance (Hedges’ *g* = -0.06, *PrI* = -0.90, 0.79). High levels of compliance were demonstrated for each exercise treatment.

**Conclusions:** Aerobic, resistance, and mind-body exercise demonstrate equivalence to mitigate symptoms of depression in older adults aged ≥ 65 years, with comparably encouraging levels of compliance to exercise treatment. These findings coalesce with previous findings in clinically depressed older adults to encourage personal preference when prescribing exercise for depressive symptoms in older adults, irrespective of severity.

**Registration:** PROSPERO CRD42018115866 (23/11/2018).

## Introduction

At the close of this decade, the last remaining ‘baby boomers’ will transition to an expanding peer demographic aged ≥ 65 years projected to constitute more than one billion older adults, worldwide ^1^. Physical exercise is proposed as a low-risk adjunctive mitigant of age-associated functional deterioration in mental health, including for dementia ^2^ and depression ^3, 4^. In light of impending demographic shifts, and with the burden of age-associated depression estimated to affect ~20% of older adults ^5– 7^, 2030 may confer a burden of 200 million adults aged ≥ 65 years presenting with clinical depression. In preparation for inevitable future demands on primary care systems ^8^, there is prevailing opportunity for concerted efforts to support primary and allied health personnel with informed preventative strategies.

International public health consortia are in concert with the antidepressant effects of exercise as a low-risk adjunct for optimal mental health ^9– 11^. While we do not yet have the answers for low uptake of exercise in older adults ^12^, it may in some way be due to nuanced regimen design, which in turn, may similarly impact compliance in exercise prescription by primary and stakeholders in aged care. By exemplar, ‘Exercise is Medicine’ is a global initiative promulgated through 40 member countries by the American College of Sports Medicine with a platform to promote and encourage routine physical exercise for general health and a broad range of medical conditions. This manifesto encourages primary care physicians and health care providers to refer patients to qualified exercise personnel when prescribing treatment plans. However, despite conventional agreement for exercise as a prophylactic for geriatric depression, contemporary literature is yet to quantify the antidepressant treatment effectiveness for individual exercise types.

Perhaps a lesser appreciated obstacle to optimising exercise prescription for mental health in older adults lies with the ‘catch all’ characterisation of exercise. During the past four decades, widely different metabolic, social, and environmental demands between exercise modalities (i.e., running vs. weightlifting vs. Tai Chi) have been well-characterised. Given that there is variation between exercise regimens, and these variations are not merely semantics, one may be surprised to discover that only a few randomised controlled trials (RCTs) have deliberately compared the antidepressant effects of different exercise regimens in older adults ^13, 14^. This begs a meaningful question, ‘are all exercise types equal?’.

In attempting to quantify the magnitude of potential antidepressant effects of exercise, researchers have deployed conventional pairwise meta-analysis ^4, 15, 16^ during recent years. In departure from the value offered by pooling conventional treatments during pairwise meta-analysis, this amalgamation is inherently prone to overgeneralisation and concomitant overestimation of treatment effectiveness ^17^. More specifically in mental health literature, pooling individual trial effect comparisons during the pairwise meta-analytical process precludes any opportunity for head-to-head comparison between different exercise types. In the same vein, a further nuance of pairwise meta-analysis is that effect-sensitivity is compromised by foregoing potentially relevant characterisation of control arms (i.e., wait-list, usual care, attention-control) of included trials ^18^. In this respect, one must acknowledge that pairwise meta-analysis has intrinsic restrictive boundaries in circumstances where head-to-head comparison of different exercise treatments are required.

In circumstances where relative effectiveness of multiple treatment comparisons are required, network meta-analysis offers a methodological solution to provide more precise pooled head-to-head effect estimates than may otherwise be achieved ^19, 20^. Performed correctly, network meta-analysis can allow comparative effectiveness of exercise treatment regimens (aerobic, resistance, and mind-body), avoiding the assumption of treatment homogeneity, controlling for bias from small study effects, and likewise, controlling for characteristically different control arms (wait-list, usual care, and attention-control).

Exercise regimens are broadly categorized into either aerobic, resistance, or mind-body exercise types ^21, 22^, and any individual exercise program may consist of multiple combinations of these activities. Extended description of these three exercise types may be found as *Extended data*. It is well established that independent regimens of either aerobic or resistance exercise elicit uniquely different metabolic and phenotypic adaptations, whereas mind-body exercise is unique as a low impact form of exercise. Therefore, it is reasonable to theorise comparative differences in the capacity to elicit any antidepressant effect. Until recently, investigation of head-to-head treatment effects of aerobic, resistance, and mind-body exercise had not been undertaken and their comparative ability to moderate symptoms of depression in older adults without pre-existing clinical depression is unknown.

Recently, Miller and colleagues ^23^ quantitatively compared head-to-head effectiveness of aerobic, resistance, and mind-body exercise in older adults with pre-existing clinical depression. In realisation of their study hypothesis, Miller *et al.* ^23^ excluded studies of older adults which lacked participant-level diagnosis of clinical depression. In doing so, there is an assumption that older adults with diagnosis of clinical depression should not be pooled with similarly aged counterparts without diagnosis. These differences are not merely semantics, and while beyond the broad scope of the present study, are worthy of summary.

Present day phenotypic characterisation of clinically depression in older adults is supported by the coalition of more than six decades of cross-disciplinary research into major depressive disorders. Limbic brain regions, monoamine neurotransmitters, and the hypothalamic-pituitary-adrenal (HPA) axis contribute to the pathophysiology of depression ^24^. In addition, hypothalamic oversight of pulsatile stress hormone perception from brain regions is central for homeostatic regulation of human stress response and maintenance of systemic feedback loop physiology in concert with the pituitary and adrenal cortex. Similarly, it is well-established that stress hormones have broad infiltration through large areas of the brain via neuronal supply originating from midbrain and brainstem regions. Indeed, these monoaminergic systems have been widely recognised by neuroscientists, pharmacologists, and clinicians alike as key determinants (thus targets) of a person’s mood, cognition, sleep quality, appetite, and reward systems; each of which is known to be affected by physical exercise and the cornerstone of many pharmacological treatments for major depressive disorders.

Long-term prospective data ^25^ has demonstrated qualitatively distinct populations within patients with major depressive disorder, and a more recent systematic review ^26^ of 67,318 participants enrolled to longitudinal cohort studies identified that people with subthreshold depression had an elevated risk of developing major depressive disorder. However, the existence for a continuum of depressive symptomology is inconclusive ^27^ and subsyndromal symptoms are not yet classified as a continuum ^28^. Taken together, it remains prudent to respect the binary threshold separating clinical diagnosis with subclinical depressive symptomology, and it stands to reason that clinical and mentally healthy categories should not be ‘merged’ into the same network in order to compare head-to-head effectiveness of different exercise treatments.

With these aspects in mind, the purpose of this systematic review and network meta-analysis was to quantitively assess the best evidence from RCTs to establish relative (head-to-head) effectiveness of resistance, aerobic, and mind-body exercise in adults aged ≥ 65 years below the clinical threshold for diagnosed depression. More specifically, we investigated whether (i) resistance, aerobic, and mind-body exercise training can induce substantive treatment effect on depressive symptoms in older adults, (ii) while considering relative treatment compliance to each exercise regimen, and further, (iii) to juxtapose the optimal exercise treatment for all adults aged ≥ 65 years irrespective of depressive symptomology.

## Methods

This review was prospectively registered with PROSPERO (registration number: CRD42018115866, 23/11/2018). The network meta-analysis extension for the Preferred Reporting Items for Systematic Reviews and Meta-Analyses (PRISMA-NMA) guidelines ^29^ and the Cochrane Intervention Review that Compares Multiple Interventions ^30^ each provided further support in guiding this review. Guidelines specific for geriatric meta-analyses ^31^ were consulted to identify baseline characteristics, equity considerations, inclusion/exclusion criteria, known confounders, and potentially important effect modifiers. *Extended data* for this review can be found online ^32^.

### Eligibility criteria

Studies were eligible for inclusion if they (i) followed an RCT protocol, (ii) used a wait-list, usual care, or attention-control group, (iii) included one or more aerobic, resistance, or mind-body exercise intervention arms, (iv) reported depressive symptoms as an outcome at baseline and during follow-up, (v) used one or more psychometrically validated depression questionnaires, (vi) recruited participants with a minimum mean sample age of 65 years. Studies were excluded when (i) the intervention condition used a multicomponent treatment including non-exercise components with the exercise condition, or (ii) the participants were diagnosed with clinical depression, defined by DSM or ICD criteria, or a clinical threshold on a questionnaire validated against a structured diagnostic interview prior to study enrollment. Eligibility was not restricted to specific years, languages, or publication status.

### Literature search

Studies were identified from computerised searches of the following databases: PubMed, Web of Science, Cumulative Index to Nursing and Allied Health Literature (CINAHL), Health Source: Nursing/Academic Edition, PsycARTICLES, PsycINFO, and SPORTDiscus. Databases were searched for key terms pertaining to four main concepts: older adults, exercise, depressive symptoms, and RCTs. Search terms were identified using text mining procedures and an example of a search strategy is presented in detail as *Extended data*. Published studies, systematic reviews, and meta-analyses ^4, 15, 16, 33– 36^ were also screened for additional articles. Databases were searched up to and including September 12 ^th^, 2019.

### Study selection

All articles collated from the systematic search initially went through a title and abstract screening, followed by a full-text screening. Eligibility criteria were used to determine whether each article should be included or excluded. The screening process was performed concurrently for both the current review and a parallel review ^23^. Once the screening process was complete, the remaining studies were included in the systematic review and descriptively reported. Studies were only included in the quantitative analysis if they contained sufficient outcome data.

### Data extraction and coding

Detailed data extraction was undertaken independently by a minimum of two researchers (KJM, PA, and/or DH) in compliance with a data extraction form (see *Extended data*), with any inconsistencies being arbitrated by another researcher (CM). Study characteristics are presented in Table 1. Methodological characteristics of each study were used to evaluate the quality of evidence, which included publication status, intention-to-treat principle, use of a cluster design, and validated measure(s) of depressive symptoms used.

**Table 1.** Participant, intervention, and methodological characteristics of studies included in the systematic review.

Author (year)	Treatment M _age_ (SD), females (%)	Control M _age_ (SD), females (%)	Source of participants	Inclusion criteria	Intention- to-treat	Cluster design	Treatment group	Control group	Length of intervention	Adherence (%)	Outcome measure(s)
Adler (2007) ^37^	72.8 (5.4) 100	70.8 (8.0) 87.5	Community	Osteoarthritis	Yes	N/R	*n* = 8 Mind-body (Tai Chi), supervised, group, low intensity, 1 time/week, 60 mins/session **Encouraged to practice at home at least 3 times per week for 15 minutes	*n* = 6 Attention- control (bingo)	10 weeks	85	CESD-20
Angeles (2016) ^38^	65.0 (3.3) 80	69.3 (7.4) 70	N/R	N/R	N/R	No	*n* = 10 Mind-body (flexibility, toning, and balance - FlexToBa DVD), supervised, group, N/R, 3 times/week, 50 mins/session	*n* = 10 Usual care	12 weeks	N/R	POMS-D
Ansai (2015) ^39^	82.8 (2.8) 65.2	82.6 (2.6) 65.2	Community	Sedentary	Yes	No	*n* = 23 Resistance (strength exercises with machines), supervised, group, N/R, 3 times/week, 60 mins/ session Adverse events: Mild muscle pain ( *n* = 9)	*n* = 23 Usual care	16 weeks	N/R	GDS-15
Antunes (2005) ^40^	68.1 (5.5) 0	65.9 (3.8) 0	Community	Sedentary	N/R	No	*n* = 23 Aerobic (ergometric cycling), supervised, N/R, moderate intensity (50-60% VO _2max_), 3 times/week, 20–60 mins/ session	*n* = 23 Wait-list	6 months	N/R	GDS-30
Bernard (2015) ^41^	65.5 (4.0) 100	65.5 (4.4) 100	Community	N/R	Yes	No	*n* = 61 Aerobic (walking - Acti’March Program), supervised, individual, moderate intensity (40-75% HR _max_), 2 times/week, 40 mins/ session **One 40 minute unsupervised session	*n* = 60 Wait-list	6 months	53.8	BDI
Bethany (2005) ^42^	83.1 (4.8) 92.9		Community	N/R	N/R	No	*n* = 11 Aerobic (chair aerobics), supervised, group, N/R, 3 times/week, 30 mins/ session	*n* = 10 Attention- control (social games)	6 weeks	71.7	BDI-II
							*n* = 10 Aerobic (walking), unsupervised, individual, N/R, 3 times/week, 30 mins/ session			62.2	
							*n* = 11 Mind-body (chair yoga), supervised, group, N/R, 3 times/week, 30 mins/ session			78.3	
Blumenthal (1991) ^43^	66.5 (4.3) 48.4	66.8 (4.3) 52.9	Community	Sedentary	N/R	No	*n* = 31 Aerobic (ergometric cycling, walking, jogging, and arm ergometry), supervised, group, moderate intensity (70% HR _max_), 3 times/week, 60 mins/session	*n* = 32 Wait-list	16 weeks	95.8	CESD-20
	67.8 (5.9) 50						*n* = 34 Mind-body (yoga), supervised, group, low intensity, 2 times/week, 60 mins/session			100	
Bonura (2014) ^44^	77.0 (7.3) 69.8		Community	N/R	N/R	No	*n* = 33 Resistance (chair strength and balance), supervised, group, N/R, 1 time/week, 45 mins/session **Practice on their own for 15 minutes during non- class days	*n* = 32 Wait-list	6 weeks	83.8	GDS-30
							*n* = 33 Mind-body (chair yoga), supervised, group, N/R, 1 time/week, 45 mins/session **Practice on their own for 15 minutes during non- class days			95	
Bostrom (2016) ^45^	84.4 (6.2) 75.3	85.9 (7.8) 76.3	Residential	Dementia	N/R	Yes (36)	*n* = 83 Resistance (functional weight-bearing and balance - High-Intensity Functional Exercise Program), supervised, group, moderate-high intensity, 2.5 times/week, 45 mins/session	*n* = 80 Attention- control (seated social activities)	4 months	73	GDS-15; MADRS *
Bouaziz (2019) ^46^	72.9 (2.5) 70	74.3 (3.4) 76.6	Community	Sedentary	N/R	No	*n* = 27 Aerobic (cycle ergometry), supervised, individual, moderate intensity, 2 times/ week, 30 mins/session	*n* = 29 Wait-list	9.5 weeks	94.7+	GADS-D
Brown (2009) ^47^	81.5 (6.9) 91.6	78.1 (6.4) 86.8	Community	N/R	Yes	Yes (8)	*n* = 26 Mind-body (flexibility and relaxation), supervised, group, low intensity, 2 times/week, 60 mins/ session	*n* = 34 Usual care	6 months	44.8	GDS-30
Cancela (2016) ^48^	80.6 (8.3) 43.8	82.9 (7.4) 81	Residential	Dementia	Yes	No	*n* = 73 Aerobic (low resistance cycling), supervised, individual, N/R, 7 times/ week, 15+ mins/session	*n* = 116 Attention- control (recreational activities)	15 months	88	CSDD
Chen (2009) ^49^	65.8 (4.3) 83.9	72.4 (6.0) 62.1	Community	N/R	N/R	Yes (8)	*n* = 62 Mind-body (silver yoga), supervised, group, N/R, 3 times/week, 70 mins/ session	*n* = 66 Wait-list	6 months	87	TDQ
Chen (2015) ^50^	79.2 (7.0) 49.1		Residential	Wheelchair- bound	N/R	Yes (10)	*n* = 59 Aerobic (resistance bands, aerobic motion, and harmonic stretching - Wheelchair-bound Senior Elastic Band Program), supervised, group, N/R, 3 times/week, 40 mins/ session	*n* = 55 Usual care	6 months	94.5	TDQ
Chen (2017) ^51^	80.7 (8.0) 58.5	81.6 (6.7) 54.8	Residential	Dementia; wheelchair- bound	N/R	Yes (8)	*n* = 65 Aerobic (resistance bands, aerobic motion, and harmonic stretching - Wheelchair-bound Senior Elastic Band Program), supervised, group, N/R, 3 times/week, 40 mins/ session	*n* = 62 Usual care	15 months	96.9	CSDD
Chin A Paw (2004) ^52^	81.0 (5.8) 73.2	81.3 (4.4) 82.9	Residential	N/R	Yes	No	*n* = 41 Resistance (progressive resistance training), supervised, group, moderate intensity, 2 times/ week, 45–60 mins/session Adverse events: Program was too intensive ( *n* = 8)	*n* = 35 Attention- control (educational programs)	6 months	78	GDS-30
Choi (2018) ^53^	77.6 (5.69) 90.9	78.8 (5.83) 96.7	Residential	N/R	N/R	Yes (6)	*n* = 33 Mind-body (sitting yoga), supervised, group, moderate intensity (10- 14/20 RPE), 4 times/week, 30-40 mins/session	*n* = 30 Wait-list	12 weeks	N/R	GDS-15
Clegg (2014) ^54^	79.4 (7.9) 73.3	78.0 (10.5) 69.2	Community	Frailty	Yes	No	*n* = 40 Resistance (strengthening exercises - Home-based Older People’s Exercise Program), unsupervised, individual, N/R, 15 times/ week, 15 mins/session Adverse events: Fell at least once ( *n* = 7)	*n* = 30 Usual care	12 weeks	67	GDS-15
Collier (1997) ^55^	71 (N/R) 63	69 (N/R) 46.2	Community	N/R	N/R	No	*n* = 25 Resistance (strength exercises with machines), supervised, group, N/R, 3 times/week, 50 mins/ session	*n* = 13 Usual care	10 weeks	N/R	GDS-30
Conradsson (2010) ^56^	85.3 (6.1) 73.6	84.2 (6.8) 72	Residential	N/R	Yes	Yes (34)	*n* = 75 Resistance (functional weight-bearing and balance - High-Intensity Functional Exercise Program), supervised, group, high intensity, 2.5 times/week, 45 mins/session	*n* = 90 Attention- control (seated social activities)	3 months	72	GDS-15
Cramer (2016) ^57^	68.7 (9.1) 37	67.8 (10.4) 40.7	Community	Colorectal cancer	Yes	No	*n* = 27 Mind-body (hatha yoga), supervised, group, N/R, 1 time/week, 90 mins/session Adverse events: Muscle soreness ( *n* = 3), abdominal pain ( *n* = 1), neck pain ( *n* = 1), minor vertigo ( *n* = 1), hip pain ( *n* = 1)	*n* = 27 Wait-list	10 weeks	53	HADS-D
De Lima (2019) ^58^	66.2 (5.5) N/R	67.2 (5.2) N/R	Community	Parkinson’s diease	N/R	No	*n* = 17 Resistance (free weights and machines), supervised, group, N/R, 2 times/week, 30-40 mins/session	*n* = 16 Usual care	20 weeks	N/R	HRSD
Donesky- Cuenco (2009) ^59^	72.2 (6.5) 73.3	67.7 (11.5) 71.4	Community	Chronic obstructive pulmonary disease	Yes	No	*n* = 14 Mind-body (yoga), supervised, group, N/R, 2 times/week, 60 mins/ session	*n* = 15 Usual care	12 weeks	83.3	CESD-20
Dong (2013) ^60^	68.5 (N/R) 100		Community	N/R	N/R	No	*n* = 32 Mind-body (YiJinJing qigong), supervised, group, N/R, 3 times/week, 60 mins/ session	*n* = 34 N/R	12 weeks	N/R	GDS-15
							*n* = 36 Mind-body (LiuZiJue qigong), supervised, group, N/R, 3 times/week, 60 mins/ session			N/R	
Dorner (2007) ^61^	86.7 (6.1) 76.7	86.9 (5.7) 76.7	Residential	Frailty; cognitive impairment	N/R	No	*n* = 15 Resistance (resistance bands, soft weights, and balance), supervised, group, N/R, 3 times/week, 50 mins/ session	*n* = 15 N/R	10 weeks	91.8	GDS-15
Emery (1990) ^62^	72 (6) 83.3		Community	N/R	N/R	No	*n* = 14 Aerobic (aerobic walking and rhythmic muscle strengthening), supervised, group, moderate intensity (70% HR _max_) 3 times/week, 60 mins/session	*n* = 13 Wait-list	12 weeks	61-94	CESD-20
								*n* = 11 Attention- control (social games)			
Eyigor (2009) ^63^	73.5 (7.6) 100	71.2 (5.5) 100	Community	N/R	N/R	N/R	*n* = 19 Aerobic (folkloric dance), supervised, group, N/R, 3 times/week, 60 mins/ session **Walk for 30 minutes at least twice a week	*n* = 18 Usual care	8 weeks	N/R	GDS-30
Eyre (2017) ^64^	68.1 (8.7) 65.8	67.6 (8.0) 65.9	Community	Cognitive impairment	N/R	No	*n* = 29 Mind-body (kundalini yoga), supervised, group, N/R, 1 time/week, 60 mins/session Adverse events: Dizziness ( *n* = 1) **Home meditation and finger movements for 12 minutes	*n* = 32 Attention- control (memory enhancement training)	12 weeks	59.4	GDS-30
Fakhari (2017) ^65^	69.2 (5.5) 48.1	69.3 (5.0) 58.6	Residential	Sedentary	N/R	No	*n* = 27 Mind-body (Tai Chi), supervised, group, N/R, 3 times/week, 20-25 mins/ session	*n* = 29 Usual care	12 weeks	N/R	BDI-II
Fransen (2007) ^66^	70.8 (6.3) 67.9	69.6 (6.1) 82.9	Community	Osteoarthritis	Yes	No	*n* = 56 Mind-body (Tai Chi), supervised, group, N/R, 2 times/week, 60 mins/ session	*n* = 41 Wait-list	12 weeks	N/R	DASS-D
Frye (2007) ^67^	69.2 (9.3) 64.3		Community	Sedentary	N/R	No	*n* = 23 Mind-body (Tai Chi), supervised, group, low- moderate intensity, 3 times/ week, 60 mins/session **Tai Chi video to assist with home practice, but was not monitored	*n* = 21 Usual care	12 weeks	72.2-100	CESD-20
Gary (2004) ^68^	67 (11) 100	69 (11) 100	Community	Diastolic heart failure	N/R	No	*n* = 15 Aerobic (walking), supervised, individual, low- moderate intensity (40–60% HR _max_), 3 times/week, 20-30 mins/session	*n* = 13 Attention- control (educational programs)	12 weeks	N/R	GDS-15
Gusi (2008) ^69^	71 (5) 100	74 (6) 100	Community	Moderate depression or overweight	N/R	No	*n* = 55 Aerobic (walking), supervised, group, N/R, 3 times/week, 50 mins/ session	*n* = 51 Usual care	6 months	N/R	GDS-15
Hars (2014) ^70^	75 (8) 97	76 (6) 95.6	Community	Risk of falling	Yes	No	*n* = 66 Aerobic (aerobic training), supervised, group, N/R, 1 time/week, 60 mins/session	*n* = 68 Wait-list	6 months	79	HADS-D
Hsu (2016) ^71^	80.7 (9.7) 63.3	81.7 (6.3) 63.3	Residential	Wheelchair- bound	Yes	No	*n* = 30 Mind-body (seated Tai Chi), supervised, group, N/R, 3 times/week, 40 mins/ session	*n* = 30 Usual care	26 weeks	85.3	GDS-15
Irwin (2012) ^72^	70.7 (5.9) 69.6	71.4 (7.7) 51.4	Community	N/R	Yes	No	*n* = 58 Mind-body (Tai Chi), supervised, group, N/R, 3 times/week, 40 mins/ session	*n* = 53 Attention- control (health education)	16 weeks	83	BDI
Irwin (2014) ^73^	66.3 (7.4) 64.6	66.4 (7.7) 72	Community	Insomnia	Yes	No	*n* = 48 Mind-body (Tai Chi), supervised, group, N/R, 1 time/week, 120 mins/ session	*n* = 25 Attention- control (sleep seminar education)	4 months	81	IDS-C
Kekalainen (2018) ^74^	68.9 (2.7) 53.8	68.3 (2.3) 47.8	Community	N/R	Yes	No	*n* = 24 Resistance (progressive resistance training), supervised, group, N/R, 2 (month 1–3) and 1 (month 4–9) times/week, 60 mins/ session	*n* = 19 Usual care	9 months	N/R	BDI-II
	67.7 (2.8) 59.6						*n* = 25 Resistance (progressive resistance training), supervised, group, N/R, 2 (month 1–3) and 2 (month 4–9) times/week, 60 mins/ session			N/R	
	69.0 (3.3) 57.1						*n* = 27 Resistance (progressive resistance training), supervised, group, N/R, 2 (month 1–3) and 3 (month 4–9) times/week, 60 mins/ session			N/R	
Kim (2019) ^75^	76.1 (3.85) 100	76.40 (3.27) 100	Community	N/R	N/R	No	*n* = 11 Resistance (strength exercises), N/R, N/R, moderate intensity (9-13/20 RPE), 3 times/week, 30–60 mins/session	*n* = 10 Usual care	24 weeks	N/R	GDS-15
Kohut (2005) ^76^	73.1 (5.6) 50	70.3 (5.6) 53.8	Community	Sedentary	N/R	No	*n* = 14 Aerobic (treadmills or ergometric cycling), supervised, group, high intensity (71% HR _max_), 3 times/week, 20–30 mins/ session	*n* = 13 Usual care	10 months	80+	GDS-30
Krishnamurthy (2007) ^77^	70.1 (8.3) 69.6	72.3 (7.4) 73.9	Residential	N/R	N/R	No	*n* = 18 Mind-body (yoga), supervised, group, N/R, 6 times/week, 75 mins/ session	*n* = 20 Wait-list	24 weeks	N/R	GDS-15
Kuo (2018) ^78^	68.93 (3.81) 80	70.38 (5.22) 85.7	Clinical	N/R	N/R	No	*n* = 15 Aerobic (stepping exercises), supervised, N/R, moderate- high intensity (13-15/20 RPE; 60-80% HR _max_), 2 times/ week, 30 mins/session	*n* = 21 Usual care	8 weeks	N/R	GDS-15
Kupecz (2001) ^79^	74.3 (6.2) 5.1	73.6 (5.9) 13	Community	N/R	N/R	No	*n* = 30 Aerobic (walking), supervised, group, low- moderate intensity, 3 times /week, 45 mins/session Adverse events: Minor medical attention ( *n* =2)	*n* = 35 Usual care	12 weeks	66	CESD-20
Li (2001) ^80^	73.2 (4.9) 76.5		Community	Sedentary	N/R	No	*n* = 40 Mind-body (Tai Chi), supervised, group, N/R, 2 times/week, 60 mins/ session **Encouraged to practice at home	*n* = 32 Wait-list	24 weeks	90	CESD-20
Lin (2007) ^81^	77.1 (7.8) 51	76.2 (7.3) 51	Community	History of falling	N/R	No	*n* = 39 Resistance (muscle strengthening and balance), supervised, individual, N/R, 0.5 times/week, 40–60 mins/ session **Instructed to practice exercises at least three times a week	*n* = 40 Attention- control (educational programs)	4 months	N/R	GDS-15
Lincoln (2011) ^82^	66.0 (7.9) 69	66.6 (7.4) 58.6	Community	Type 2 diabetes	N/R	No	*n* = 29 Resistance (progressive resistance training), supervised, N/R, high intensity, 3 times/week, 45 mins/session	*n* = 29 Attention- control (regular phone calls)	16 weeks	N/R	GDS-30
Ma (2018) ^83^	70.2 (10.3) 31.7	69.7 (10.8) 30.4	Community	Hypertension	Yes	No	*n* = 79 Mind-body (Tai Chi), supervised, group, N/R, 4 times/week, 60 mins/ session	*n* = 79 Usual care	6 months	N/R	CESD-10
Maki (2012) ^84^	71.9 (4.1) 69.3	72.0 (3.9) 72	Community	N/R	Yes	No	*n* = 66 Aerobic (walking and group work), supervised, group, N/R, 1 time/week, 90 mins/ session	*n* = 67 Attention- control (educational programs)	3 months	87.5	GDS-30
Martinez (2015) ^85^	76.1 (8.1) 89.7	72.5 (8.0) 86.2	Clinical	N/R	Yes	No	*n* = 29 Mind-body (qigong), supervised, group, N/R, 2 times/week, 90 mins/ session	*n* = 25 Usual care	4 weeks	88.8	GDS-5
Martins (2011) ^13^	75.9 (7.0) 66.7	77.7 (8.8) 58.1	Residential	Sedentary	N/R	No	*n* = 24 Aerobic (rhythmic walking and stepping sequences), supervised, group, low-high intensity (40-85% HR _max_), 3 times/week, 45 mins/ session	*n* = 31 Usual care	16 weeks	N/R	POMS-SF-D
	73.4 (6.4) 56.5						*n* = 23 Resistance (resistance bands and calisthenics), supervised, group, moderate intensity, 3 times/ week, N/R			N/R	
McMurdo (1993) ^86^	82.3 (6.9) 80	79.3 (6.2) 80.7	Residential	N/R	N/R	N/R	*n* = 10 Resistance (strengthening exercises), supervised, group, low intensity, 2 times/week, 45 mins/ session	*n* = 23 Attention- control (reminiscence sessions)	7 months	91	GDS-30
Monga (2005) ^87^	68 (4.2) 0	70.6 (5.3) 0	Community	Prostate Cancer	N/R	No	*n* = 11 Aerobic (treadmill walking), supervised, group, moderate intensity (65% HR _max_), 3 times/week, 45–50 mins/session	*n* = 10 Usual care	8 weeks	N/R	BDI
Mortazavi (2012) ^88^	71.7 (8.2) 64.1	71.7 (8.2) 61.3	Community	N/R	N/R	No	*n* = 181 Aerobic (upper, lower, and whole body movements), supervised, group, low intensity, 2 times/week, 45 mins/session **Pamphlet explaining details of each session for exercising at home	*n* = 191 Attention- control (physical activity pamphlets)	2 months	N/R	GHQ-D
Netz (1994) ^89^	70.7 (6.8) N/R	74.1 (8.9) N/R	Clinical	Cognitive or affective deterioration	N/R	No	*n* = 15 Aerobic (light callisthenic and rhythmic movements), supervised, group, low intensity, 3 times/week, 45 mins/session	*n* = 15 Attention- control (social intellectual activity)	8 weeks	N/R	GDS-30
Ng (2017) ^90^	70.3 (5.3) 56.2	70.1 (5.0) 56	Community	Frailty	Yes	No	*n* = 48 Resistance (functional strength and balance), supervised, group, moderate intensity, 2 times/ week, 90 mins/session **Weeks 13-24 were instructed to participate at home	*n* = 50 Usual care	24 weeks	85	GDS-15
Oken (2006) ^91^	73.6 (5.1) 78.7	71.2 (4.4) 75	Community	Low activity (aerobic exercise less than 210 minutes per week)	N/R	No	*n* = 38 Aerobic (walking), supervised, group, moderate intensity (6-7/10 RPE; 70% HR _max_), 1 time/ week, 60 mins/session Adverse events: Hip pain ( *n* = 1) **Exercise daily at least 5 times per week was strongly encouraged	*n* = 42 Wait-list	6 months	68.7	CESD-10 *; POMS-D
	71.5 (4.9) 70.5						*n* = 38 Mind-body (hatha yoga), supervised, group, N/R, 1 time/week, 90 mins/session Adverse events: Groin muscle strain ( *n* = 1) **Exercise daily at least 5 times per week was strongly encouraged			77.6	
Park (2016) ^92^	75.3 (7.5) 75		Community	Osteoarthritis	N/R	No	*n* = 52 Mind-body (chair yoga), supervised, group, N/R, 2 times/week, 45 mins/ session	*n* = 48 Attention- control (health education)	8 weeks	95	PROMIS- EDD SF-8a
Payne (2008) ^93^	64.7 (6.3) 100		Community	Breast cancer	N/R	N/R	*n* = 9 Aerobic (walking), unsupervised, individual, moderate intensity, 4 times/ week, 20 mins/session	*n* = 9 Usual care	12 weeks	N/R	CESD-20
Pedersen (2017) ^94^	79.2 (6.6) 52.6	81.3 (5.1) 50	Residential	N/R	N/R	N/R	*n* = 19 Resistance (resistance training), supervised, group, N/R, 2 times/week, 60 mins/ session	*n* = 12 N/R	12 weeks	N/R	HADS-D
Penninx (2002) ^14^	68.7 (5.6) 70.1		Community	Osteoarthritis	Yes	N/R	*n* = 149 Aerobic (walking), supervised, N/R, moderate intensity (50-70% HR _max_), 3 times/week, 60 mins/ session **Months 4-18 were unsupervised	*n* = 144 Attention- control (health education)	18 months	N/R	CESD-6
							*n* = 146 Resistance (dumbbell and cuff weight exercises), supervised, N/R, N/R, 3 times/week, 60 mins/ session ** Months 4-18 were unsupervised			N/R	
Pinniger (2013) ^95^	79.4 (N/R) 100		Community	Age-related macular degeneration	N/R	No	*n* = 8 Aerobic (tango dance), supervised, group, N/R, 2 times/week, 90 mins/ session	*n* = 9 Wait-list	4 weeks	96.9	GDS-15
Ramanathan (2017) ^96^	68.9 (7.6) 100	68.2 (8.8) 100	Residential	N/R	N/R	No	*n* = 20 Mind-body (yoga), supervised, group, N/R, 2 times/week, 60 mins/ session	*n* = 20 Wait-list	12 weeks	N/R	HRSD
Roswiyani (2019) ^97^	71.90 (8.57) 70.1	74.31 (9.57) 66.2	Residential	N/R	Yes	No	*n* = 67 Mind-body (qigong), supervised, group, N/R, 2 times/week, 90 mins/ session	*n* = 65 Usual care	8 weeks	61.2	BDI-II
Sahin (2018) ^98^	84.5 (4.8) N/R	85.4 (4.7) N/R	Residential	Frailty	N/R	No	*n* = 16 Resistance (dumbbell and cuff weight exercises), supervised, N/R, low intensity (40% 1R _max_), 3 times/week, 40 mins/ session	*n* = 16 Usual care	8 weeks	N/R	GDS-15
	84.2 (6.9) N/R						*n* = 16 Resistance (dumbbell and cuff weight exercises), supervised, N/R, high intensity (70% 1R _max_), 3 times/week, 40 mins/ session			N/R	
Sattin (2005) ^99^	80.4 (3.1) 95.4	80.5 (3.2) 93.6	Residential	Frailty; history of falling	Yes	Yes (20)	*n* = 158 Mind-body (Tai Chi), supervised, group, N/R, 2 times/week, 60-90 mins/ session	*n* = 153 Attention- control (health education)	48 weeks	76	CESD-20
Sola-Serrabou (2019) ^100^	71.9 (5.0) 76.5	74.8 (6.1) 77.8	Residential	Sedentary	N/R	No	*n* = 18 Resistance (strength exercises), supervised, N/R, moderate intensity (5-6/10 RPE), 2 times/week, 60 mins/session	*n* = 17 Usual care	24 weeks	90	GDS-15
Song (2019) ^101^	76.22 (5.76) 80	75.33 (6.78) 70	Community	Cognitive impairment; low activity (aerobic exercise less than 150 minutes per week)	Yes	No	*n* = 60 Aerobic (stepping exercises), supervised, group, moderate intensity (12- 14/20 RPE), 3 times/week, 60 mins/session	*n* = 60 Attention- control (health education)	16 weeks	73.1	GDS-30
Sparrow (2011) ^102^	70.3 (7.5) 32.7	71.7 (7.2) 29.4	Community	Low activity (exercise less than 20 minutes per week)	Yes	N/R	*n* = 49 Resistance (resistance training - Telephone-Linked Computer-based Long- term Interactive Fitness Trainer), unsupervised, N/R, N/R, 3 times/week, 60 mins/session Adverse events: Musculoskeletal injury and discomfort ( *n* = 8)	*n* = 51 Attention- control (health education)	12 months	54	BDI-II
Swoap (1994) ^103^	65.2 (4.2) 53.8	65.2 (4.2) 50	Community	Sedentary	N/R	No	*n* = 24 Aerobic (walking), supervised, group, moderate intensity (65-70% HR _max_), 3 times/week, 30–65 mins/session	*n* = 11 Wait-list	26 weeks	N/R	GDS-30
	65.2 (4.2) 58.3						*n* = 14 Aerobic (walking), supervised, group, high intensity (80-85% HR _max_), 3 times/week, 30–60 mins/ session			N/R	
Tapps (2013) ^104^	76 (N/R) N/R		Residential	N/R	N/R	No	*n* = 11 Resistance (resistance band and chair exercises), supervised, group, N/R, 3 times/week, 30 mins/ session	*n* = 14 Usual care	12 weeks	N/R	BDI-II
Taylor-Piliae (2014) ^105^	71.5 (10.3) 35.8	68.2 (10.3) 52.1	Community	History of stroke	Yes	No	*n* = 53 Mind-body (Yang-style Tai Chi), supervised, group, N/R, 3 times/week, 60 mins/ session	*n* = 48 Usual care	12 weeks	82	CESD-20
Tsang (2003) ^106^	72.9 (9.5) 62.5	76.3 (8.4) 37.5	Residential	Chronic medical illness	N/R	No	*n* = 24 Mind-body (eight-section brocades qigong), supervised, group, N/R, 2 times/week, 60 mins/ session **Practice daily for at least 30 minutes	*n* = 26 Attention- control (remedial rehabilitation activities)	12 weeks	N/R	GDS-30
Tsang (2013) ^107^	83.3 (6.3) 77	84.9 (6.0) 72.7	Clinical	Frailty	Yes	No	*n* = 61 Mind-body (Yan Chai Yi Jin ten-section brocades qigong), supervised, group, N/R, 2 times/week, 60 mins/ session	*n* = 55 Attention- control (newspaper reading)	12 weeks	N/R	GDS-15
Tsutsumi (1998) ^108^	68.5 (6.1) 100		Community	Sedentary	N/R	N/R	*n* = 12 Resistance (strength exercises with machines), supervised, group, moderate intensity (55-65% 1R _max_), 3 times/week, N/R	*n* = 12 N/R	12 weeks	N/R	POMS-D
							*n* = 12 Resistance (strength exercises with machines), supervised, group, high intensity (75-85% 1R _max_), 3 times/week, N/R			N/R	
Vahlberg (2017) ^109^	72.6 (5.5) 20.6	73.7 (5.3) 27.3	Community	History of stroke	Yes	No	*n* = 34 Resistance (functional weight-bearing and balance - High-Intensity Functional Exercise Program), supervised, group, high intensity, 2 times/week, 55 mins/session	*n* = 33 Usual care	3 months	91	GDS-20
Vankova (2014) ^110^	83.4 (8.2) 91.6	82.9 (7.9) 92.4	Residential	N/R	N/R	No	*n* = 79 Aerobic (ballroom dance - Exercise Dance for Seniors Program), supervised, group, N/R, 1 time/week, 60 mins/session	*n* = 83 Wait-list	3 months	84.6	GDS-15
Wang (2009) ^111^	75.5 (9.2) 100	74.5 (8.1) 80	Community	N/R	N/R	No	*n* = 7 Mind-body (yoga), supervised, group, N/R, 2 times/week, 60 mins/ session	*n* = 10 Attention- control (social group)	4 weeks	78.1	CESD-10
Witham (2005) ^112^	80 (6) 37	81 (4) 54	Community	Frailty; chronic heart failure	N/R	No	*n* = 36 Aerobic (progressive aerobic seated exercise with small weights), supervised, group, N/R, 2 times/week, 20 mins/ session **Months 4–6 were home exercise 2–3 times per week with no face-to-face contact	*n* = 32 Usual care	6 months	82.7	HADS-D
Yang (2005) ^113^	72.6 (5.4) 68.4	72.7 (7.5) 90.5	Community	Chronic pain	N/R	No	*n* = 19 Mind-body (Chun Soo Energy Healing qigong), supervised, group, N/R, 2 times/week, 20 mins/ session	*n* = 21 Wait-list	4 weeks	N/R	POMS-D
Yeh (2003) ^114^	65 (6) 40	66 (6) 40	Community	Chronic obstructive pulmonary disease	Yes	N/R	*n* = 5 Mind-body (Tai Chi), supervised, group, N/R, 2 times/week, 60 mins/ session	*n* = 5 Usual care	12 weeks	74.2	CESD-20
Zanuso (2012) ^115^	74.3 (3.5) 50	67.1 (2.0) 50	Community	Sedentary	N/R	No	*n* = 10 Resistance (resistance training), supervised, N/R, moderate intensity, 3 times/ week, 60 mins/session	*n* = 10 Wait-list	12 weeks	90.7	POMS-D

*Note*. N/R = not reported; *n* = Total participants included in intention-to-treat or post-treatment data analysis; HR _max_ = Heart rate maximum; VO _2max_ = Maximal oxygen uptake; 1R _max_ = One-repetition maximum; RPE = Rating of perceived exertion; BDI = Beck Depression Inventory; CESD = Center for Epidemiological Studies Depression Scale; CSDD = Cornell Scale for Depression in Dementia; DASS-D = Depression, Anxiety, and Stress Scale (depression subscale); GADS-D = Goldberg Anxiety and Depression Scale (depression subscale); GDS = Geriatric Depression Scale; GHQ-D = General Health Questionnaire (depression subscale); HADS-D = Hospital Anxiety and Depression Scale (depression subscale); HRSD = Hamilton Rating Scale for Depression; IDS-C = Inventory of Depression Symptomatology; MADRS = Montgomery-Asberg Depression Rating Scale; POMS-D = Profile of Mood States (depression subscale); PROMIS-EDD SF-8a = Patient Reported Outcome Measurement Information System - Emotional Distress and Depression Short Form-8a; TDQ = Taiwanese Depression Questionnaire. *Outcome measure used in network meta-analysis. **Additional unsupervised exercise component.

Control groups within each individual study were further categorised as either wait-list, usual care, or attention-control. Participants undergoing wait-list conditions received the exercise intervention following trial completion. Participants randomised to usual care were those in sole receipt of conventional treatment during the trial. Participants randomised to attention-control conditions (also known as attention placebo control or active control) received activities completely unrelated to physical activity and/or exercise during their respective trials (e.g., social activities, educational programs, etc.).

Important participant and intervention characteristics were used to verify whether potential effect modifiers were similarly distributed across comparisons within the network. Participant characteristics were coded according to sample size, age (mean and standard deviation), percentage of females, and place of residence (community-dwelling, residential care, clinical setting). Relevant inclusion criteria (e.g., sedentary, dementia, etc.) were further used to assess the risk of bias from equity considerations ^116^.

Intervention characteristics were appropriately coded. Exercise types were categorised as aerobic, resistance, or mind-body exercise. Length of interventions were coded in weeks or months. Exercise intensities were coded according to ratings of perceived exertion ^117^, heart rate maximum (HR _max_; low = <50%, moderate = 50-70%, high = >70%), maximal oxygen uptake (VO _2max_; low = <40%, moderate = 40-60%, high = >60%) or one-repetition maximum (1R _max_; low = <50%, moderate = 50-74%, high = >74%) ^118^, or where unavailable, this was estimated according to the assessment of the original author(s). Frequency was coded as total sessions per week. Duration was coded as the average number of minutes engaging in exercise per session. Format of program was dichotomously coded as exercise in a group or individual setting. Format of supervision was dichotomously coded as supervised or unsupervised exercise. Agreement between the three researchers was 91.3%.

### Risk of bias and quality assessment

Risk of bias of the included RCTs was assessed using the Cochrane Collaboration’s Tool for Assessing Risk of Bias ^119^, which were independently conducted by a minimum of two researchers (KJM, PA, and/or DH). Discrepancies were arbitrated by another co-author (CM). Appraisal for ‘other sources of bias’ were evaluated with consideration for small sample size ( *n* < 15), low adherence (less than 80%), cluster randomisation, and inequity in the selection of the sample.

### Summary of outcomes

Outcome statistics including means ( *M*), standard deviations ( *SD*), and sample sizes ( *n*) for depressive symptoms were used to calculate the mean change in the primary outcome. Test statistics (i.e., *t*-, *F*-, and *p*-values) were used to estimate effect sizes when descriptive statistics were unavailable. Pairwise relative (head-to-head) treatment effects for depressive symptoms were estimated using Hedges’ *g* ^120^, which corrects for overestimation biases due to small sample sizes ^121^. Hedges’ *g* coefficients were interpreted according to Cohen’s conversions ^122^, whereby effects were considered small (0.2), medium (0.5), and large (0.8). Independent subgroups (e.g., males vs. females) within studies were treated as independent effect size estimates ^123^. When individual studies reported more than one post-treatment depression score for the same group of participants, only the initial post-treatment time-point was used. When studies reported depression scores on multiple outcome measures, the included depression measure was selected in compliance with clinical applicability ^124, 125^.

Secondary outcome data were also extracted for study attrition, treatment adherence, and adverse events. Pairwise relative (head-to-head) treatment effects for study attrition were reported as odds ratios based on the pre-treatment sample size versus post-treatment dropout in the treatment and comparison conditions. Treatment adherence were reported as a percentage of total attendance in, or compliance to, the treatment condition. Adverse events were qualitatively reported according to the descriptive information provided in the transcript.

### Data synthesis

One notable assumption of network meta-analysis relates to comparison group estimates, which are derived from pooling studies with homogenous between-study effect modifiers ^126^. If the distribution of an effect modifier is heterogeneous across studies within a specific comparison group, the assumption of transitivity can be violated. In the context of this network meta-analysis, we separate exercise conditions (i.e., aerobic, resistance, mind-body) and control conditions (i.e., wait-list, usual care, attention-control) to account for the between-study variation of these effect modifiers. Given the intricacies of depression severity, a forest plot was generated to depict the juxtaposition of treatment effects between (i) participants in the present network meta-analysis with depressive symptomology but not clinically diagnosed and (ii) participants in a previous network meta-analysis with clinical depression ^23^.

Risk of bias assessment identified the potential for unit-of-analysis error within studies using a cluster design for treatment allocation. Thus, sample sizes were recalculated to account for error in cluster RCTs by determining a design effect ^127^ with a conservative intracluster correlation coefficient of 0.05, in accordance with past studies ^45, 128^. The certainty of evidence contributing to network estimates was assessed according to the Grading of Recommendations Assessment, Development and Evaluation (GRADE) approach ^129^.

Data were analysed and figures were generated using STATA/SE 15.1 ^130^. Comparison-adjusted funnel plots were used to evaluate publication bias and small-study effects. Random-effects meta-regression was used to investigate modifying effects of age, gender, source of participants, length of intervention, exercise intensity, frequency, duration, and format of program, which have been identified as potential risks to transitivity in geriatric meta-analyses ^4, 35, 131^.

A multivariate random-effects meta-analysis was conducted using the ‘mvmeta’ command ^132^. A random-effects model assumes variance both within and between studies, explaining the heterogeneity of treatment effects ^133^. A common heterogeneity parameter was assumed across comparisons. Heterogeneity was evaluated using tau-squared (τ ^2^), which estimates the deviation in effect sizes across the population of studies ^123^. The 95% prediction interval ( *PrI*) was also used to estimate the true dispersion of effect within two standard deviations of the mean effect size ^123^. The significance level was *p* < 0.05 for all analyses.

## Results

### Study selection

**Figure 1.**
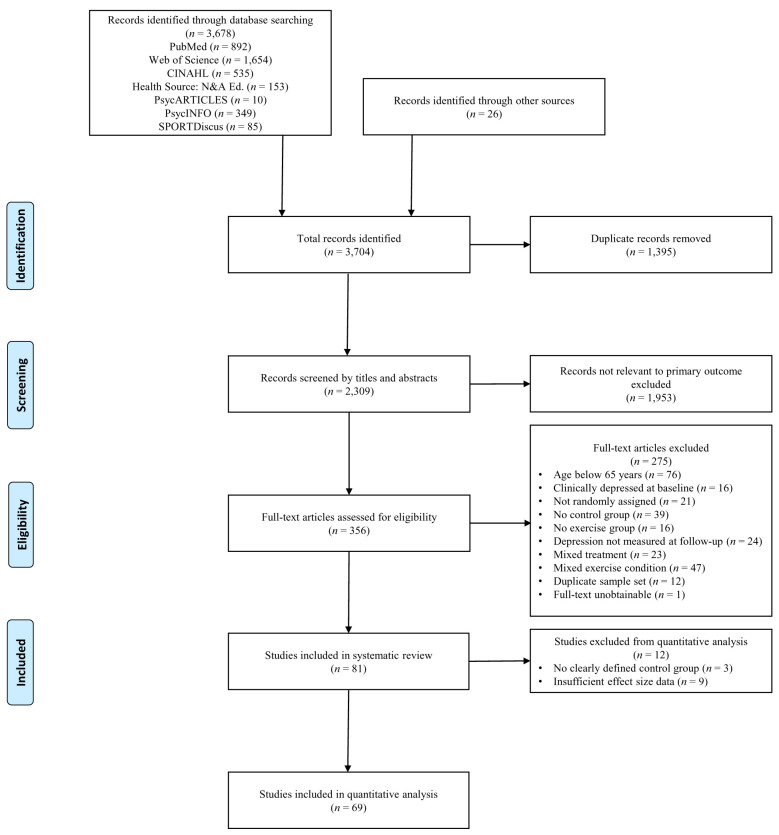
Flowchart of screening process.

The initial systematic search yielded a total of 3,704 citations. When duplicate studies were removed ( *n* = 1,395), 2,309 eligible records were identified. Titles and abstracts were screened by two independent researchers (KJM and DCGB) with an agreement of 91.9%. Subsequently, 356 full-text articles were assessed for compliance with inclusion criteria and outcome data. Full-text screening was performed independently by two researchers (KJM and PA) with an agreement of 81.8%, where discrepancies were arbitrated and resolved by consensus with a third researcher (CM). Studies using duplicate sample sets ( *n* = 12) were identified, where the most informative publication with complete data being included in the quantitative analysis. Finally, 81 studies fulfilled all inclusion criteria to be included in the systematic review. In cases of missing data, authors were emailed by the first author (KJM). If authors failed to respond following two contact efforts, and there were insufficient data to calculate effect size estimates, the study was excluded from the quantitative analysis ( *n* = 9). Additionally, three studies ^60– 62^ fulfilled all criteria but necessitated exclusion from the quantitative analysis due to control conditions being insufficiently defined, and the authors being unobtainable. Figure 1 outlines study selection and additional exclusion criteria.

### Characteristics of the studies

**Figure 2.**
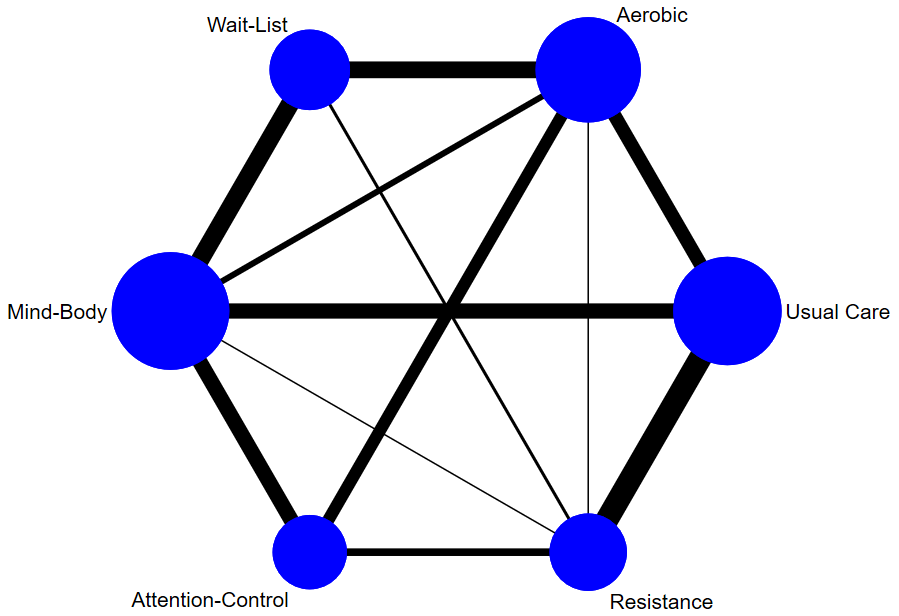
Network plot of comparisons for all studies included in the network meta-analysis. Line width is proportional to the number of pairwise effect size estimates and node size is proportional to the number of participants.

Data from a total of 5,379 participants (2,815 treatment and 2,564 control) across 69 studies were pooled in the quantitative analysis. Each study reported pre- and post-treatment measures of depression symptoms, and data from 60 of the 69 studies were obtained for post-treatment attrition. Figure 2 illustrates the network of pairwise comparisons across all studies. Aerobic, resistance, and mind-body conditions had one or more direct comparison with each of the five comparison conditions. Direct comparisons for depressive symptoms were robustly characterised between intervention and corresponding control conditions (aerobic = 28, resistance = 22, mind-body = 32), with proportions being similar for study attrition (aerobic = 24, resistance = 21, mind-body = 25).

From the 69 included studies, 43 studies enrolled young-old samples (65–75 years) and 26 studies enrolled old-old samples (>75 years). Participants were identified as either community-dwelling ( *n* = 46), living in residential care ( *n* = 18), clinical ( *n* = 4), or uncategorised ( *n* = 1). Twelve studies enrolled participants with sedentary lifestyles, while the remainder enrolled participants with a range of active and sedentary lifestyles. Some studies recruited older adults that were either frail ( *n* = 5), wheelchair bound ( *n* = 3), or identified as presenting with a risk of falling ( *n* = 2). Participants with age-associated comorbidities were also recruited, including cardiovascular problems ( *n* = 6), dementia ( *n* = 3), cognitive impairment ( *n* = 3), cancer ( *n* = 2), and other chronic illness ( *n* = 6).

From 79 independent exercise interventions, the majority were supervised in group exercise classes ( *n* = 64). The remainder were either individually supervised ( *n* = 5), unsupervised in an individual format ( *n* = 2), supervised in an undisclosed format ( *n* = 1), or entirely undisclosed ( *n* = 1). The average exercise program length (weeks) was shorter for mind-body ( *M* = 13.67, *SD* = 6.77) than aerobic ( *M* = 19.76, *SD* = 14.44) or resistance ( *M* = 18.00, *SD* = 9.07). Average weekly exercise minutes (calculated as frequency multiplied by duration) were calculated for aerobic ( *M* = 107.78, *SD* = 40.88), resistance ( *M* = 126.90, *SD* = 50.28), and mind-body exercise ( *M* = 132.75, *SD* = 76.07). Treatment adherence ( *n* = 44) was comparable between aerobic ( *M* = 81.01, *SD* = 13.72), resistance ( *M* = 81.17, *SD* = 9.00), and mind-body exercise ( *M* = 79.34, *SD* = 14.73). Adverse events were observed during several of the included trials, which are presented in Table 2.

**Table 2.** Adverse events from included studies.

Exercise group	Study	Adverse event(s)
**Aerobic**	Kupecz (2001) ^79^	Minor medical attention ( *n* = 2)
	Oken (2006) ^91^	Hip pain ( *n* = 1)
**Resistance**	Ansai (2015) ^39^	Mild muscle pain ( *n* = 9)
	Chin A Paw (2004) ^52^	Program was too intensive ( *n* = 8)
	Clegg (2014) ^54^	Fell at least once ( *n* = 7)
**Mind-body**	Cramer (2016) ^57^	Muscle soreness ( *n* = 3), abdominal pain ( *n* = 1), neck pain ( *n* = 1), minor vertigo ( *n* = 1), hip pain ( *n* = 1)
	Eyre (2017) ^64^	Dizziness ( *n* = 1)
	Oken (2006) ^91^	Groin muscle strain ( *n* = 1)

### Risk of bias and quality assessment

**Figure 3.**
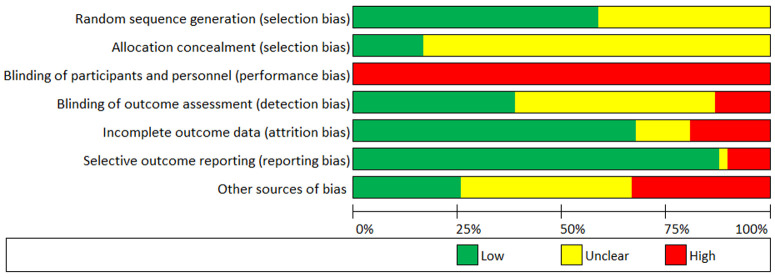
Risk of bias chart of studies included in the quantitative analysis.

Risk of bias for each study is presented comprehensively as *Extended data* (see *Figure S1*). Both blinding of participants and personnel to treatment was implausible due to the implicit nature of exercise training interventions, and thus, the remaining six criteria were used to assess the overall risk of bias within each study. Methodological quality of included studies can be considered low-to-moderate ( *M* = 4.08/6, where low = 1, unclear, = 0.5, high = 0). Assessment of random sequence generation (selection bias), incomplete outcome data (attrition bias), and selective outcome reporting (reporting bias) may be considered adequate for most studies, whereas allocation concealment (selection bias) and blinding of outcome assessment (detection bias) were diverse. High ‘other sources of bias’ was due to low study adherence ( *n* = 14), small sample sizes ( *n* = 8), or both ( *n* = 1). See Figure 3 for a summary of the risk of bias in the included studies.

### Assessment of inconsistency

Inconsistency network models were used to test the global consistency of direct and indirect effects of pairwise and multi-arm comparisons. Assumption of consistency was satisfied for each treatment ( *p* > 0.05). Tests for inconsistency between direct and indirect estimates were not significant ( *p* > 0.05), thus indirect and direct estimates were not different to direct evidence. Inconsistency tables can be found as *Extended data* (see *Tables S1* and *S2*).

Loop-specific heterogeneity was explored using inconsistency plots (see *Extended data, Figure S7* and *S8*). Within the depressive symptoms network, inconsistency factors ( *IF*) did not indicate high inconsistency ( *IF* = 0.00 to 0.65) or loop-specific heterogeneity (τ ^2^ = 0.05 to 0.28). The AER-MB-UC loop departed from the minimum lower-bound confidence interval ( *CI*), yet fell short of presenting risk for heterogeneity ( *IF* = 0.54, τ ^2^ = 0.05, *CI* = 0.04, 1.03). Within the attrition network, the ratio of two odds ratios ( *RoR*) indicated a high degree of inconsistency for the AER-RES-UC ( *RoR* = 1.87) and MB-RES-UC ( *RoR* = 1.77) loops. Each were interpreted as presenting elevated risk for heterogeneity, which were subsequently downgraded during GRADE assessment. All remaining loops satisfied assumption of consistency.

### Publication bias and sensitivity analyses

**Figure 4.**
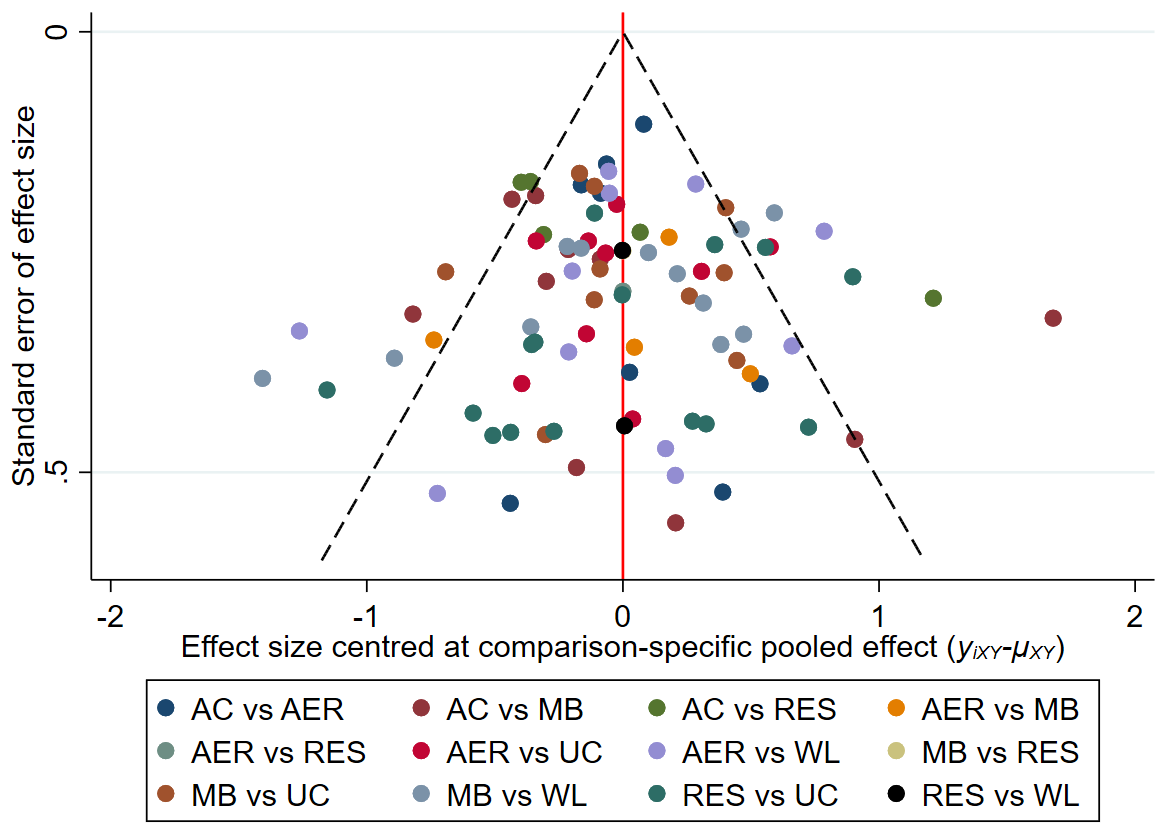
Comparison-adjusted funnel plot for the depressive symptoms network. The red vertical line represents the null hypothesis that independent effect size estimates are not different to comparison-specific pooled estimates. AC = attention-control; AER = aerobic; MB = mind-body; RES = resistance; UC = usual care; WL = wait-list.

Comparison-adjusted funnel plots were used to detect publication bias and small-study effects. Funnel plots were roughly symmetrical for both depressive symptoms and attrition, indicative of low risk of publication bias and no presence of small-study effects. See Figure 4 for the depressive symptoms network and *Extended data* for the attrition network (see *Figure S9*).

In order to test transitivity across networks, potential effect modifiers were tested with meta-regression sensitivity analyses for the entire pool of studies and separately for each exercise comparison (aerobic vs. resistance vs. mind-body). No significant modifying effects were observed for the pool of studies or any separate exercise comparison for age, gender, source of participants, length of intervention, format of program, exercise intensity, frequency, duration, adherence, year of study, risk of bias, publication status, intention-to-treat analysis, nor cluster design, indicating that the assumption of transitivity was upheld. Full analyses are presented as *Extended data* (see *Tables S6*– *S8*).

### Results of the network meta-analysis

Data pooled from 69 eligible studies provided a total of 88 individual comparisons for depressive symptoms and 76 individual comparisons for study attrition. Table 3 presents the network meta-analysis of depressive symptoms and attrition. Network estimates were calculated to establish relative effectiveness between pairs of comparisons.

**Table 3.** League table for head-to-head comparisons.

**Wait-list**	-0.11 (-0.40, 0.17)	-0.45 (-0.77, -0.13)	-0.51 (-0.74, -0.27)	-0.39 (-0.63, -0.14)	-0.12 (-0.41, 0.18)
1.10 (0.48, 2.48)	**Usual care**	-0.34 (-0.57, -0.10)	-0.39 (-0.62, -0.16)	-0.27 (-0.51, -0.04)	-0.00 (-0.27, 0.27)
0.74 (0.30, 1.83)	0.68 (0.37, 1.26)	**Resistance**	-0.06 (-0.33, 0.22)	0.06 (-0.22, 0.35)	0.33 (0.05, 0.61)
1.00 (0.50, 2.01)	0.92 (0.47, 1.77)	1.35 (0.61, 2.99)	**Mind-body**	0.12 (-0.12, 0.35)	0.39 (0.15, 0.63)
1.03 (0.50, 2.14)	0.94 (0.48, 1.84)	1.39 (0.62, 3.10)	1.03 (0.51, 2.07)	**Aerobic**	0.27 (0.02, 0.52)
1.03 (0.41, 2.57)	0.94 (0.41, 2.12)	1.38 (0.60, 3.19)	1.02 (0.47, 2.23)	1.00 (0.46, 2.15)	**Attention-control**

*Note*. Depressive symptoms (upper) are reported as Hedges’ *g* and 95% confidence intervals. Negative scores indicate a greater decrease in depressive symptoms for the column group. Attrition (lower) is reported as odds ratios and 95% confidence intervals. An odds ratio lower than 1 indicate a greater attrition for the column-defining comparison.

**Figure 5.**
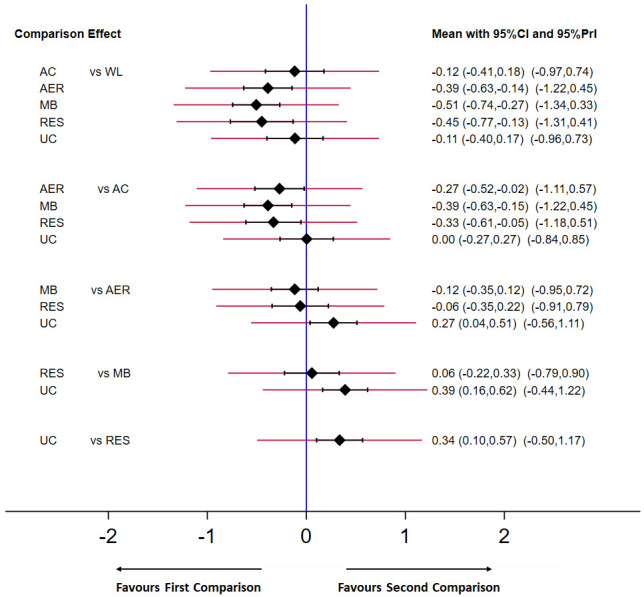
Predictive interval plot for the depressive symptoms network. Black diamonds represent the difference in the effect size estimate (Hedges’ *g*). Narrow horizontal lines represent the confidence intervals ( *CI*) and the wider horizontal lines represent the prediction intervals ( *PrI*). The blue vertical line represents the null hypothesis (Hedges’ *g* = 0). Negative scores indicate a greater decrease in depressive symptoms for the comparison (left) group. AC = attention-control; AER = aerobic; MB = mind-body; RES = resistance; UC = usual care; WL = wait-list.

Each exercise type effectively reduced depressive symptoms compared with control conditions (see Figure 5). Ranking of treatments for depressive symptoms are presented on SUCRA plots of ranked mean values, which can be found as *Extended data* (see *Figure S11* and *Table S3*). Ranked order of quantitative values determined mind-body exercise to be the most effective type of exercise to mitigate depressive symptoms, followed closely by resistance and aerobic exercise, respectively. The magnitude of study effect did not reach statistical threshold to favour any individual exercise treatment.

Resistance exercise demonstrated the highest study compliance compared with each of the other comparison groups, but the dispersion of effect estimates presented a level of heterogeneity that confounded any substantive differences. Comprehensive reporting of study attrition can be found as *Extended data* (see *Figure S10*) in addition to SUCRA plots and ranked order (see *Figure S12* and *Table S4*).

### Effectiveness vs. attrition

**Figure 6.**
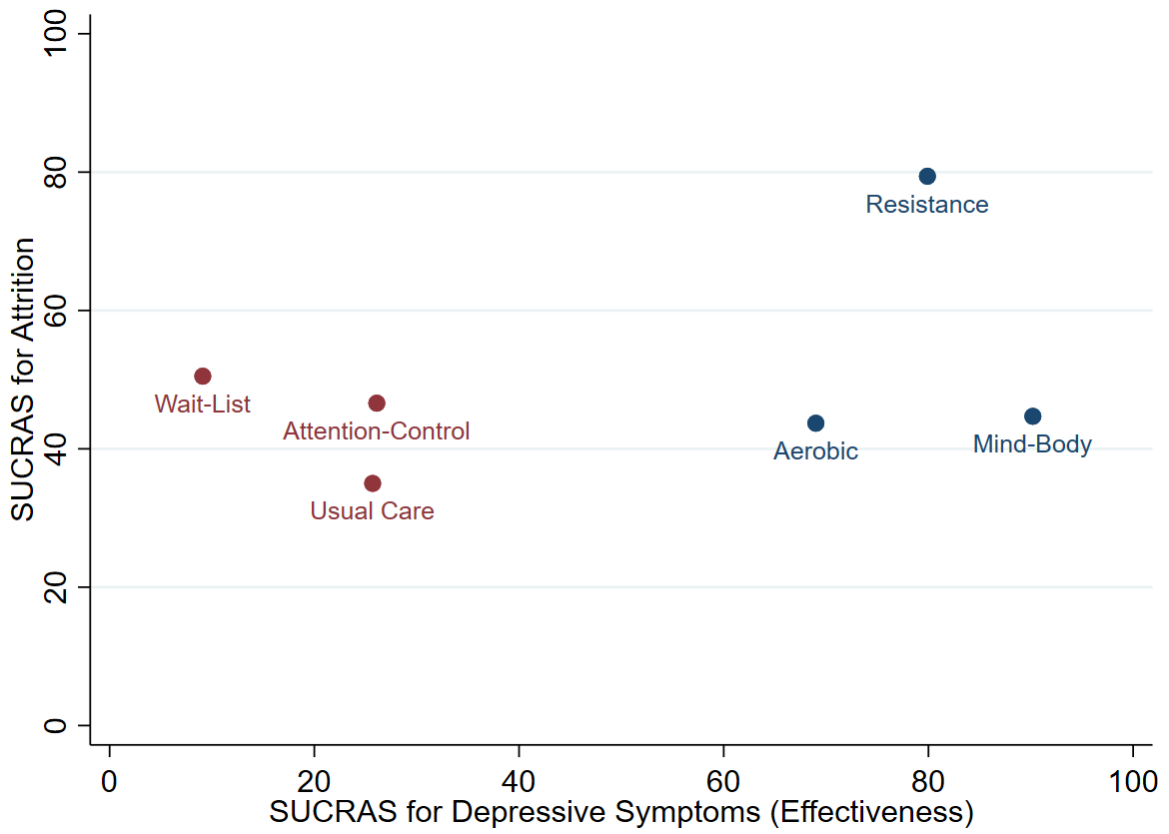
Clustered ranking plot of the network presenting clustered analysis of SUCRA values for depressive symptoms (effectiveness) and attrition outcomes. Each colour represents a group of treatments that belong to the same cluster. Treatments in the upper right corner represent effective treatments with low attrition.

A two-dimensional clustered ranking plot was employed to illustrate the average reduction in depressive symptoms for each comparison, relative to average attrition rate. Figure 6 presents the ranking of the exercise conditions with respective control conditions in conjunction with SUCRA values for depressive symptoms (effectiveness) and attrition. The three exercise conditions amalgamated a single cluster and were more effective than control conditions.

### GRADE assessment

The certainty of evidence was assessed with the GRADE approach ^129^. All control comparisons in the depressive symptoms network were rated as high or moderate certainty, which were downgraded due to small sample size or potential risks of bias (i.e., attrition bias, detection bias, or bias resulting from low adherence). Comparisons between exercise interventions were moderate-to-low certainty, resulting from imprecision in confidence intervals and small sample sizes. Detailed summary of the depressive symptoms network is presented in Table 4. Estimates of the attrition network were downgraded due to inconsistency and imprecision, which reflect moderate to very low confidence this outcome (see *Extended data*, *Table S5*).

**Table 4.** Summary of GRADE assessment for the certainty in depressive symptoms estimates.

Comparison Effect	Number of participants	Number of direct comparisons	Nature of evidence	Certainty	Reason for downgrading
**Aerobic vs. wait-list**	371 vs. 357	11	Mixed	High	None
**Aerobic vs. usual care**	309 vs. 310	9	Mixed	Moderate	Risk of bias ^a^
**Aerobic vs. attention-control**	431 vs. 472	8	Mixed	High	None
**Resistance vs. wait-list**	43 vs. 42	2	Mixed	Moderate	Imprecision ^b^
**Resistance vs. usual care**	358 vs. 272	15	Mixed	High	None
**Resistance vs. attention-control**	267 vs. 274	5	Mixed	Moderate	Risk of bias ^c^
**Mind-body vs. wait-list**	380 vs. 363	12	Mixed	High	None
**Mind-body vs. usual care**	358 vs. 356	10	Mixed	Moderate	Risk of bias ^d^
**Mind-body vs. attention-control**	298 vs. 265	10	Mixed	High	None
**Aerobic vs. resistance**	24 vs. 23	1	Mixed	Low	Imprecision ^b e^
**Aerobic vs. mind-body**	90 vs. 83	4	Mixed	Moderate	Imprecision ^e^
**Resistance vs. mind-body**	33 vs. 33	1	Mixed	Low	Imprecision ^b e^

^a^Potential attrition bias due to high number of studies with incomplete outcome data. ^b^Small sample size. ^c^Potential risk of bias due to high number of studies with low adherence. ^d^Potential detection bias due to high number of studies without blinding of outcome assessment. ^e^Confidence intervals include values favouring either treatment.

### Generalisation for all adults aged ≥ 65 years

**Figure 7.**
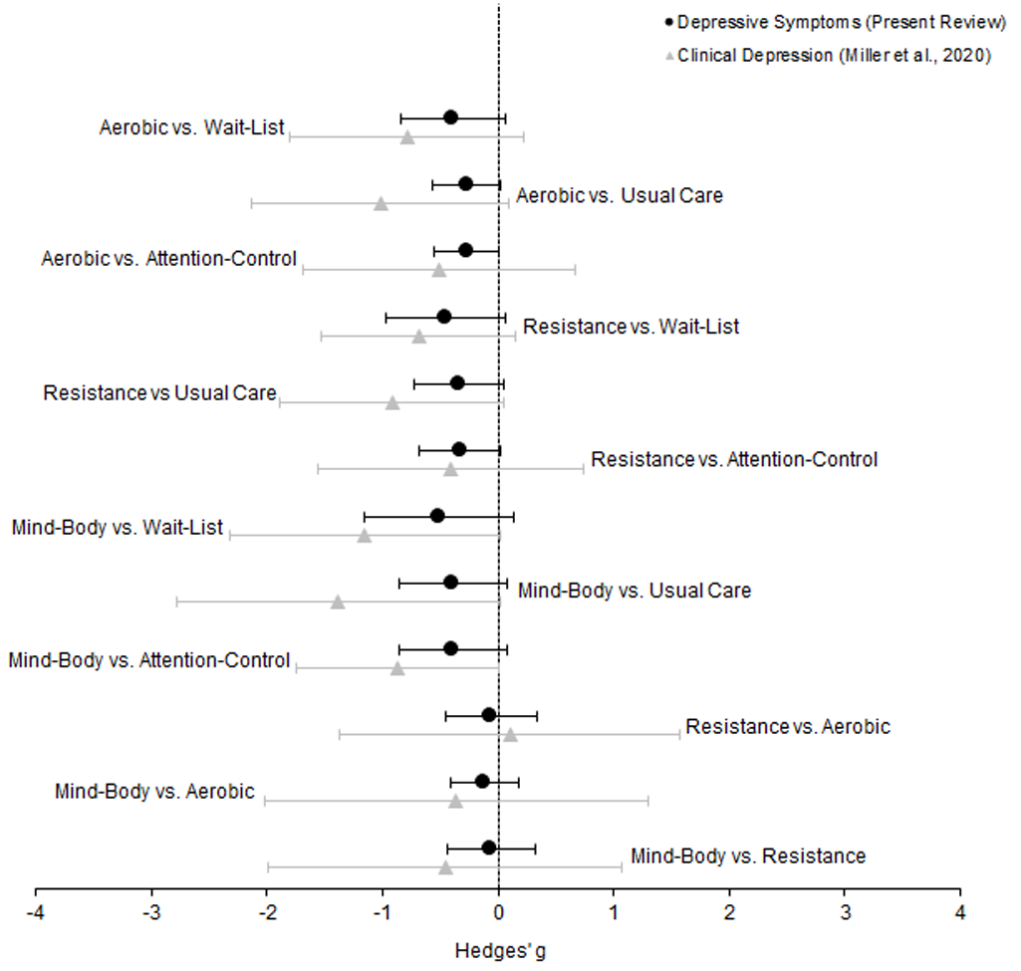
Forest plot depicting juxtaposed collective RCT evidence of older adults ( *n* = 5,975) in the depressive symptoms network. Circles represent the difference in the effect size estimate (Hedges’ *g*) for those with depressive symptomology but not diagnosed with clinical depression ( *n* = 5,379). Triangles represent the difference in the effect size estimate (Hedges’ *g*) for those with clinical depression ( *n* = 596; effect sizes adapted from Miller *et al.*, 2020). Horizontal lines represent the confidence intervals ( *CI*). The dotted vertical line represents the null hypothesis (Hedges’ *g* = 0). Negative scores indicate a greater decrease in depressive symptoms for the left group, and vice versa.

Figure 7 presents collective representation for all adults aged ≥ 65 years. This comparison employs scaled distribution (Hedges’ *g*) for the present data and that of clinically depressed older adults ^23^.

## Discussion

The present review offers new information for general exercise prescription to support mental health outcomes for adults aged ≥ 65 years. Specifically, (i) aerobic, resistance, and mind-body each demonstrate equivalent benefit to mitigate symptoms of depression in adults aged ≥ 65 years, (ii) compliance to exercise treatment is notably encouraging for each exercise types, and (iii) when combined with the pool of data from clinically depressed older adults ^23^, the effectiveness of aerobic, resistance, and mind-body exercise is comparably consistent for all adults aged ≥ 65 years, irrespective of depression severity. These findings should provide reassurance for personnel and stakeholders in healthy ageing to encourage exercise prescription from a point of pragmatism and in collaboration with patient preference.

### Theoretical implications for the current findings

Behavioural and physiological research ^24, 25, 27, 28^ coalesce to provide ample reasoning to separate participant cohorts with existing clinical depression from those without diagnosis. This study is no different. In agreement with findings from previous research ^13, 14, 35^, aerobic and resistance exercise demonstrated similar treatment effectiveness in older samples aged ≥ 65 years (Hedges’ *g* = -0.06, *PrI* = -0.91, 0.79). Exercise characteristics (i.e., intensity, frequency, duration, etc.) are often similar between aerobic and resistance exercise, representing two sides of the same coin. Meta-analytical data ^15^ on older adults with existing clinical depression observed that exercise programs incorporating a combination of aerobic and resistance training were most beneficial. Although the synergistic effects of combined exercise types were beyond the scope of the current review, it is conceivable that aerobic and resistance exercise may complement one another in an exercise intervention.

Pooled direct and indirect estimates marginally favoured treatment with mind-body exercise over either aerobic (Hedges’ *g* = -0.12, *PrI* = -0.95, 0.72) or resistance exercise (Hedges’ *g* = -0.06, *PrI* = -0.90, 0.79). However, it must be noted that the magnitude of effect falls short of being statistically different between groups and should be considered equivalent. Certainty of evidence is moderate, due to the dispersion in effect size estimates resulting in imprecision. Direct comparisons from multi-arm RCTs have offered mind-body exercise to be more effective than aerobic ^91^ or resistance ^44^ exercise. Moreover, subgroup analyses ^16^ have indicated that clinically depressed older adults respond more favourably to mind-body exercise, but this hypothesis has not be substantiated ^4^.

Since mind-body exercise engages low intensity muscular activity (i.e., yoga, Tai Chi, qigong), the novel evidence in the current systematic analysis challenge the idea that intensity is the primary mechanism for the antidepressive effect of exercise. Rather, mind-body exercise combines the mental and physical aspects of exercise, which may result in similar antidepressive effects to higher intensity exercise ^134, 135^. Critical to these mental aspects is interoceptive sensations such as an internally directed focus on breathing and proprioception, which have previously been linked to the resilience of depressive states ^136, 137^. Thus, it is plausible that mind-body exercise allows older adults to regulate negative mood states, which is not normally possible during aerobic and resistance activities.

Other important determinants of successful programming include study attrition, adherence rate, and adverse events. Here, we hypothesised that each exercise condition would demonstrate lower compliance to treatment than wait-list, usual care, and attention-control comparisons. Contrary to expectations, pooled direct and indirect estimates indicated that study attrition was comparable for all comparisons apart from resistance exercise, which offered a higher degree of compliance. However, on deeper scrutiny of absolute sample size, any differences observed in attrition become abrogated due to relatively smaller participant numbers within the resistance exercise studies ( *n* = 705) compared with aerobic ( *n* = 1,143) or mind-body ( *n* = 1,005). Thus, in consideration of the wide dispersion in effect size estimates and a moderate risk of bias in individual studies, there are limitations in the certainty to confidently conclude substantive difference in study attrition between any comparisons.

Each of the three types of exercise had similar adherence rates and time spent per week (calculated as frequency multiplied by duration). Interestingly, mind-body exercise had relatively shorter intervention length than either aerobic or resistance exercise ( *M*_diff_ = 6.09 and 4.33 weeks, respectively) despite having a greater reduction in depressive symptoms. This could be explained by (i) mind-body exercise having the same antidepressive effects in a shorter time than aerobic and resistance exercise, or (ii) a potential plateau effect whereby the antidepressive effects reach a maximum threshold during the first 10–15 weeks of an exercise program and are then maintained during the remaining weeks. Either way, it seems plausible that mind-body exercise provides a slightly more effective intervention against depressive symptoms in older populations without clinical depression, but that these treatment effects are not substantive enough to constitute statistical difference.

### Practical considerations

With consideration to projected population estimates over the next decade and the consequential demand on healthcare services ^1, 8^, the findings from this network meta-analysis offer a message of support for exercise prescription to promote mentally healthy ageing. When considering the collective findings of the present review in conjunction with the recent network meta-analysis in clinically depressed adults aged ≥ 65 years ^23^, stakeholders in healthy ageing and exercise prescription have encouraging pooled RCT evidence for the antidepressant effects of either aerobic, resistance, or mind-body exercise for older adults across the mental health continuum.

Treatment safety is a matter of ongoing importance in gerontological health, and exercise treatment programs are no different. Systematic scrutiny of the included RCTs found that study participants reported no major adverse events and only a few minor somatic complaints ( *n* = 28). Taken together, this provides encouraging support for personnel wanting to safely prescribe exercise-based intervention programs in older populations. Of course, there is always a possibility of underreporting adverse events in clinical trials, and the present review was no exception. Therefore, the importance of reporting event outcomes, adverse or otherwise, cannot be understated. In fact, there is a known phenomenon in geriatric exercise research whereby adverse events are often underreported because authors do not consider minor adverse events to be noteworthy and/or essential to the primary purpose of the trial ^138^, giving rise to an ongoing issue that will not be corrected until all studies routinely report event outcomes.

Nevertheless, participants engaging in aerobic exercise reported the least adverse events ( *n* = 3), including minor medical attention and hip pain. Amongst studies included in this meta-analysis, aerobic exercise predominantly involved walking and stationary cycling, which may reflect a safe and natural form of exercise for older adults. Resistance exercise was typically associated with participants experiencing mild muscular pain and falls ( *n* = 16), which may be explained by the progressive overloading of resistance-based training. Notably, incidents of falling were reported in an unsupervised exercise program. Finally, mind-body exercise was typically associated with different types of muscular pain and body strain ( *n* = 9). It is speculated that the higher rates of injury in mind-body exercise are predominantly because it incorporates flexibility, balance, and stability movements, which may be unique to older bodies. In general, exercise seems to be a relatively safe intervention for older adults living in both the local community and residential aged care, although intensity and supervision, particularly for resistance training, should be monitored to ensure falls and injury do not occur.

The present review has some notable advantages above a traditional pairwise meta-analysis. RCTs with considerable non-exercising components, such as those using a multicomponent exercise intervention, were excluded because they may have overestimated the magnitude of the true effect in past reviews. Specifically, it is likely that multicomponent exercise interventions such as laughter therapy ^139^, depression awareness training ^128^, or self-efficacy training ^140^ may have introduced a risk of bias by inflating the observed effectiveness of the exercise program on depressive symptoms through a secondary, complementary treatment effect. Pairwise meta-analysis also assumes that all control groups are the same, which is not always the case ^18^. To manage heterogeneity from this assumption, control groups were separated into individual network comparisons. Taken together, the current findings provide a more accurate estimate of the true effects of exercise on depressive symptoms in adults aged ≥ 65 years.

### Limitations and future directions

The present network meta-analysis is not without limitations. As study participants and personnel cannot be successfully blinded, there is an inherent risk of performance bias. It is also believed that many exercise-based interventions have a small number of participants, shorter follow-up, and do not adequately conceal randomisation ^141^, which are all likely to reduce the quality of RCTs and increase the risk of bias. However, we mitigated the impact of this by comparing relative effects with multiple control groups in order to increase reliability and specificity. This, combined with the relatively low risk of bias in individual RCTs, were extremely important in minimising overall risk of bias and achieving accurate effect comparisons in the present review.

Since most RCTs did not explicitly describe the exclusion of participants with ongoing diagnosis of clinical depression, there was potential contamination with data from participants with existing clinical diagnosis and medical treatment that went unreported. This was primarily managed by separating (i) participants with depressive symptomology but not clinically diagnosed in the present review from (ii) participants with clinical depression in a previous network meta-analysis ^23^. Within this review, we further mitigated this effect modifier by only including RCTs, where this risk would be balanced by control participants. We recommended that ageing researchers encourage the reporting of all ongoing pharmacological regimens in trials recruiting older participants.

Although modifying effects were explored using meta-regression, potential compounding effects from exercise modifiers (e.g., fitness improvements, length of program, session frequency and duration, exercise intensity, supervision, group format) were outside the scope of our network meta-analysis. There has been a modicum of such exploration in subgroup and meta-regression analyses of previous reviews ^4, 16, 35^, providing researchers with an encouraging opportunity in their planning of future similar work. Future meta-analyses with extensive subgroup analyses should explain the heterogeneity of effect sizes between similar exercise intervention studies in older persons.

## Conclusions

Pooled RCT evidence highlights that each individual exercise mode (aerobic, resistance, and mind-body) demonstrate equivalence to mitigate symptoms of depression in older adults, irrespective of depression severity. As each exercise treatment demonstrated encouraging levels of treatment compliance, we endorse personnel and stakeholders in healthy ageing to encourage individual/patient preference when prescribing exercise to older adults ≥ 65 years presenting with depressive symptomology.

## Data availability

### Underlying data

Figshare: Aerobic, resistance, and mind-body exercise are equivalent to mitigate symptoms of depression in older adults: A systematic review and network meta-analysis of randomised controlled trials (extended data). https://doi.org/10.6084/m9.figshare.12998549.v2 ^32^.

This project contains the following underlying data:

- Data File D1 (PDF file containing raw outcome data)
- STATA/SE 15.1 files (DTA files containing raw data)

### Extended data

Figshare: Aerobic, resistance, and mind-body exercise are equivalent to mitigate symptoms of depression in older adults: A systematic review and network meta-analysis of randomised controlled trials (extended data). https://doi.org/10.6084/m9.figshare.12998549.v2 ^32^.

This project contains the following extended data:

- Supplementary File S1 (PDF file containing additional information, tables, and figures not in the main manuscript)

### Reporting guidelines

Figshare: PRISMA-NMA checklist for “Aerobic, resistance, and mind-body exercise are equivalent to mitigate symptoms of depression in older adults: A systematic review and network meta-analysis of randomised controlled trials (extended data). https://doi.org/10.6084/m9.figshare.12998549.v2 ^32^

Data are available under the terms of the Creative Commons Attribution 4.0 International license (CC-BY 4.0).
