# Supplementary material for: Aerobic, resistance, and mind-body exercise are equivalent to mitigate symptoms of depression in older adults: A systematic review and network meta-analysis of randomised controlled trials
Source: F1000Res. 2021 Jul 14;9:1325. Originally published 2020 Nov 13. [Version 2] doi: 10.12688/f1000research.27123.2 (PMC8191520; doi:10.12688/f1000research.27123.2)
Supplement: Peer review history

# Peer review: Reviewer response for version 2

**DOI:** 10.5256/f1000research.58270.r89650

**Published:** 2021-07-26

**Authors:** Andrea Camaz Deslandes

**Affiliations:** 1 Federal University of Rio de Janeiro, Rio de Janeiro, Brazil

The manuscript has improved substantially, and the authors addressed my’ comments satisfactorily, improving the manuscript’s quality. I have no further comments to make.

Are the rationale for, and objectives of, the Systematic Review clearly stated?

Yes

Is the statistical analysis and its interpretation appropriate?

Yes

Are sufficient details of the methods and analysis provided to allow replication by others?

Yes

Are the conclusions drawn adequately supported by the results presented in the review?

Partly

Reviewer Expertise:

Physical exercise and mental health

I confirm that I have read this submission and believe that I have an appropriate level of expertise to confirm that it is of an acceptable scientific standard.

# Peer review: Reviewer response for version 2

**DOI:** 10.5256/f1000research.58270.r89648

**Published:** 2021-07-15

**Authors:** Walid Kamal Abdelbasset

**Affiliations:** 1 Department of Health and Rehabilitation Sciences, College of Applied Medical Sciences, Prince Sattam bin Abdulaziz University, Al-Kharj, Saudi Arabia; 2 Department of Physical Therapy, Kasr Al-Aini Hospital, Cairo University, Cairo, Egypt

Appreciating the authors for their responses to all required comments. I have no additional comments. Congrats

Are the rationale for, and objectives of, the Systematic Review clearly stated?

Partly

Is the statistical analysis and its interpretation appropriate?

Yes

Are sufficient details of the methods and analysis provided to allow replication by others?

Yes

Are the conclusions drawn adequately supported by the results presented in the review?

Partly

Reviewer Expertise:

Exercise Intervention; Geriatric Rehabilitation; Rehabilitation Sciences

I confirm that I have read this submission and believe that I have an appropriate level of expertise to confirm that it is of an acceptable scientific standard.

# Peer review: Reviewer response for version 1

**DOI:** 10.5256/f1000research.29961.r83975

**Published:** 2021-06-09

**Authors:** Christian Imboden

**Affiliations:** 1 Private Clinic Wyss AG, Sierre, Switzerland; 2 Psychiatric Services Solothurn, University of Basel, Basel, Switzerland

**General**

 The review and network meta-analysis are very well written and conclusive. I especially appreciate the aim of the authors to analyze studies of different modalities of physical activity (PA) on depressive symptoms in older adults without clinical depression and comparing them to their already published network-MA on the effects of PA in clinically depressed older adults. The methods are described thoroughly. The authors analyze RCTs evaluating the effects of aerobic, resistance or mind-body exercise on depressive symptoms separately. In addition, they also group control groups by waitlist, attention control and care as usual which yields a very detailed analysis. They found significant positive effects on depressive symptoms for all three modalities of PA versus control interventions with no specific modality yielding significantly stronger effects than the other. They also analyzed attrition to the study protocol, an important variable that adds additional weight to the analysis. Considering attrition, the authors found comparable values in all comparisons. The results are displayed in appropriate ways by graphical and numerical means. And thus, provides a good overview of the various results.

 The statistical methods and interpretation seem absolutely appropriate to me, but since I am not a specialist for network meta-analysis I can not provide an in-depth evaluation.

**I have some minor comments to the authors:**

**P 24, Results of the network-MA:**

 Please spell out “SUCRA” at its first mentioning

**Figure 7:**

 In the notes, the confidence interval is not specified. Is it 95% CI? Please specify. I also don’t understand why the horizontal lines (95%? CI) are different from Figure 5: i.e., the comparison AER vs. WL has a 95% CI of -0.63 to -0.14. So why does the horizontal line depicting the CI cross the vertical zero-line in Figure 7? Please explain.

**Discussion:**

 p. 27: you described that mind-body exercise has additional mental effects such as interoceptive sensations. Often mind-body exercise also incorporates aspects of mindfulness. Therefore, I believe it would be appropriate to insert a short paragraph discussing the effects of mindfulness (such as meditation and MBSR) on depressive symptoms. A suggested reference might be: Li *et al.* (2019)  DOI: 10.1111/inm.12568 ^1^

 On the same page, you discuss compliance to treatment. You state, that you hypothesized that compliance would be lower in exercise treatments. However, the meta-analysis on dropout rates of Stubbs *et al.* (2016), DOI: 10.1016/j.jad.2015.10.019 ^2^ found similar dropout rates for exercise and control conditions but analyzed samples of adult patients (not specifically elderly). Nonetheless, you might discuss this in a sentence.

**Conclusions:**

 You state that exercise mitigates symptoms of depression regardless of depression severity. If interpreted strictly, according to the current paper this only applies to subclinical severity of symptoms. If you incorporate your earlier paper (network MA of exercise in clinically depressed elderly) into the conclusion, I believe this should be mentioned accordingly.

Are the rationale for, and objectives of, the Systematic Review clearly stated?

Yes

Is the statistical analysis and its interpretation appropriate?

I cannot comment. A qualified statistician is required.

Are sufficient details of the methods and analysis provided to allow replication by others?

Yes

Are the conclusions drawn adequately supported by the results presented in the review?

Yes

Reviewer Expertise:

Depression, physical activity for depression

I confirm that I have read this submission and believe that I have an appropriate level of expertise to confirm that it is of an acceptable scientific standard.

## References

[1] : The effects of mindfulness-based stress reduction on depression, anxiety, and stress in older adults: A systematic review and meta-analysis. *Int J Ment Health Nurs* .2019;28(3) : 10.1111/inm.12568 635-656 10.1111/inm.12568 30656813

[2] : Dropout from exercise randomized controlled trials among people with depression: A meta-analysis and meta regression. *J Affect Disord* .2016;190: 10.1016/j.jad.2015.10.019 457-466 10.1016/j.jad.2015.10.019 26551405

# Response

**Published:** 2021-06-30

**Authors:** Kyle Miller

**Affiliations:** Federation University, Australia

***General***

***The review and network meta-analysis are very well written and conclusive. I especially appreciate the aim of the authors to analyze studies of different modalities of physical activity (PA) on depressive symptoms in older adults without clinical depression and comparing them to their already published network-MA on the effects of PA in clinically depressed older adults. The methods are described thoroughly. The authors analyze RCTs evaluating the effects of aerobic, resistance or mind-body exercise on depressive symptoms separately. In addition, they also group control groups by waitlist, attention control and care as usual which yields a very detailed analysis. They found significant positive effects on depressive symptoms for all three modalities of PA versus control interventions with no specific modality yielding significantly stronger effects than the other. They also analyzed attrition to the study protocol, an important variable that adds additional weight to the analysis. Considering attrition, the authors found comparable values in all comparisons. The results are displayed in appropriate ways by graphical and numerical means. And thus, provides a good overview of the various results.***

***The statistical methods and interpretation seem absolutely appropriate to me, but since I am not a specialist for network meta-analysis I can not provide an in-depth evaluation.***

 We wish to firstly thank the Reviewer for their thoughtful commentary. We have taken time to consider all feedback and make the necessary revisions to the article.

***I have some minor comments to the authors:***

***P 24, Results of the network-MA:***

***Please spell out “SUCRA” at its first mentioning***

 We have included “surface under the cumulative ranking curve” before the first mention of the acronym.

***Figure 7:***

***In the notes, the confidence interval is not specified. Is it 95% CI? Please specify.***

 We thankful to the Reviewer for picking up this clerical error, on our behalf. This has now been rectified to state the “95% CI” in notes for Figures 5 and 7.

***I also don’t understand why the horizontal lines (95%? CI) are different from Figure 5: i.e., the comparison AER vs. WL has a 95% CI of -0.63 to -0.14. So why does the horizontal line depicting the CI cross the vertical zero-line in Figure 7? Please explain.***

 We are grateful to the Reviewer for highlighting a lack of consistency in the article, specifically for intervals between Figure 5 and 7. Version 1 of the article as read by Reviewers contained both confidence and prediction intervals for Figures 5 and 7, respectively. In light of this, Figure 7 is now converted to 95% confidence intervals in order to maintain observational consistency with Figure 5. We have also proofed consistency of data (and presentation thereof) within article figures.

***Discussion:***

***p. 27: you described that mind-body exercise has additional mental effects such as interoceptive sensations. Often mind-body exercise also incorporates aspects of mindfulness. Therefore, I believe it would be appropriate to insert a short paragraph discussing the effects of mindfulness (such as meditation and MBSR) on depressive symptoms. A suggested reference might be: Li et al. (2019)  DOI: 10.1111/inm.125681***

 This is a very reasonable query relating to the potential for ‘mindfulness’ being an adjunct to potential improvement from mind-body exercise. The authors have taken the opportunity to record these findings within the article, with enhanced detail within the online supplementary material. This supplementary material extends upon the current article discussion to include potential discrete mechanisms that may underpin the psycho-adaptive response to exercise in older adults. This can be found within the online repository which accompanies this article:

- ‘Data Availability’ > ‘Extended data’ > ‘Supplementary File S1’
- https://doi.org/10.6084/m9.figshare.12998549.v3

***On the same page, you discuss compliance to treatment. You state, that you hypothesized that compliance would be lower in exercise treatments. However, the meta-analysis on dropout rates of Stubbs et al. (2016), DOI: 10.1016/j.jad.2015.10.0192 found similar dropout rates for exercise and control conditions but analyzed samples of adult patients (not specifically elderly). Nonetheless, you might discuss this in a sentence.***

 This is an interesting point and one which we credited with consideration during the planning of this review. As the Reviewer will be aware, every epidemiological study conducted to date identify older adults to be the least active and least compliant with exercise compared with younger demographics. For reasons of being conservative and containing the potential for observer bias, this was handled in the negative unless data demonstrated otherwise. However, on reflection we deem the use of ‘hypothesised’ as potentially inappropriate compared with alternatives such as ‘anticipated’. This has been amended in the Discussion section to read: “Here, we anticipated that each exercise condition would demonstrate lower compliance to treatment than wait-list, usual care, and attention-control comparisons.”

***Conclusions:***

***You state that exercise mitigates symptoms of depression regardless of depression severity. If interpreted strictly, according to the current paper this only applies to subclinical severity of symptoms. If you incorporate your earlier paper (network MA of exercise in clinically depressed elderly) into the conclusion, I believe this should be mentioned accordingly.***

 We extend our gratitude to this Reviewer, who in coalition with Reviewer 2, identified this very same point. On reflection, this was clearly an overreach by the authors. We fully accept the requirement for amendment, which resulted in amendments to the Abstract, Discussion, and Conclusion sections.

# Peer review: Reviewer response for version 1

**DOI:** 10.5256/f1000research.29961.r83980

**Published:** 2021-05-24

**Authors:** Andrea Camaz Deslandes

**Affiliations:** 1 Federal University of Rio de Janeiro, Rio de Janeiro, Brazil

**Objective:**

 The study aimed to investigate the head-to-head effectiveness of aerobic, resistance, and mind-body exercise in ameliorating depressive symptoms in older adults. The study is relevant and original, considering the increased prevalence of depression in older adults and the need for treatment through complementary and alternative practices, such as exercise. This topic has indeed received little attention in literature until now. The method used is adequate to the purpose of the study, and the results and the discussion were developed appropriately. However, some minor adjustments are necessary for a better understanding of the manuscript. Please find below some of the specific items regarding the issues.

**Introduction:**

 I suggest a presentation of the concepts of physical activity and exercise, as well as a discussion about divergences in the classification of Yoga and other mind-body practices as physical exercise.

**Methods:**

 The inclusion of older adults with Dementia (n=3), Cognitive Decline (n=3), Parkinson's Disease (n=1), and Cognitive frailty (n=1) can be a possible limitation of the present study, considering the differences related to comorbidity. I suggest the author exclude these studies. Neuropsychiatric symptoms in MCI and Dementia are related to several different mechanisms, as well as the psychological and physiological effects of physical exercise in these populations. The characteristics of Depression in these cases can be very specific, with causes, neurophysiological changes and treatments that differ from depressive symptoms in healthy older adults. The inclusion of these studies can contribute of shrinking the effect size of exercise. For example, the effectiveness of pharmacological approaches to managing depressive symptoms in Dementia and MCI is less effective and the antidepressant effect of exercise in these populations is still controversial. If the authors decide to include these population (MCI, Parkinson, Dementia), several other studies that investigated the effect of exercise on neuropsychiatric symptoms (such as depression) in patients with MCI and Dementia should be included in the present meta-analysis, For more details, see Liang 2018 and Leng *et al.*, 2018. ^1^ Moreover, this issue should be included in the limitation section.  

 Factors related to the prescription of exercise (frequency, intensity, duration, length, supervision, cognitive engagement) should be more explored in analyses and results.

**Discussion:**

 The author should discuss the possible different mechanisms related to the antidepressant effect of mind-body practices, aerobic and resistance training in older adults, considering both psychological and physiological hypotheses. Please consider more recent neurobiological mechanisms related to the effect of exercise on depressive symptoms (e.g., myokines, neurotrophins, inflammatory cytokines, etc.).

**Conclusion:**

 The authors concluded: “Pooled RCT evidence highlights that each individual exercise mode (aerobic, resistance, and mind-body) can effectively mitigate symptoms of depression in older adults, irrespective of depression severity”. I suggest excluding the phrase “ ***irrespective of depression severity***”, since the authors did not run a specific analysis to evaluate this issue.

Are the rationale for, and objectives of, the Systematic Review clearly stated?

Yes

Is the statistical analysis and its interpretation appropriate?

Yes

Are sufficient details of the methods and analysis provided to allow replication by others?

Yes

Are the conclusions drawn adequately supported by the results presented in the review?

Partly

Reviewer Expertise:

Physical exercise and mental health

I confirm that I have read this submission and believe that I have an appropriate level of expertise to confirm that it is of an acceptable scientific standard, however I have significant reservations, as outlined above.

## References

[1] : Effects of Physical Exercise on Depressive Symptoms in Patients With Cognitive Impairment: A Systematic Review and Meta-Analysis. *J Nerv Ment Dis* .206(10) : 10.1097/NMD.0000000000000887 809-823 10.1097/NMD.0000000000000887 30273278

# Response

**Published:** 2021-06-30

**Authors:** Kyle Miller

**Affiliations:** Federation University, Australia

***Objective:***

***The study aimed to investigate the head-to-head effectiveness of aerobic, resistance, and mind-body exercise in ameliorating depressive symptoms in older adults. The study is relevant and original, considering the increased prevalence of depression in older adults and the need for treatment through complementary and alternative practices, such as exercise. This topic has indeed received little attention in literature until now. The method used is adequate to the purpose of the study, and the results and the discussion were developed appropriately. However, some minor adjustments are necessary for a better understanding of the manuscript. Please find below some of the specific items regarding the issues.***

 Firstly, the authors are extremely grateful for the considered commentary from this Reviewer. We take this opportunity to extend our gratitude for being provided an opportunity to improve our article. The author team have taken time to deliberate on each point and have reached consensus within our responses below. Thank you.

***Introduction:***

***I suggest a presentation of the concepts of physical activity and exercise, as well as a discussion about divergences in the classification of Yoga and other mind-body practices as physical exercise.***

 We acknowledge this as an important point, and one which would require detailed expansion and integration of broad knowledge of the different forms of exercise classification and their nuanced differences. In order to preserve the flow of rationale within the Introduction section, while similarly being complete with exercise characteristics of aerobic, resistance, and mind-body regimens, we have provided supplementary reading to satisfy the broader readership of exercise specialists, including background material for readers out-with this specialist topic.

 We have taken this opportunity to include further statement within the Introduction section to highlight the differences between types of exercise: “We direct the reader to the Extended data of this article for detailed description of the exercise classifications included in this review.”

 ‘Extended data’ for publication in F1000 is repository for non-essential information or data. To diligently tackle important divergences in classifications in exercise type required 750 words, which the authors deemed as an important point with an appropriate home within the online repository, which can be found here:

- ‘Data Availability’ > ‘Extended data’ > ‘Supplementary File S1’
- https://doi.org/10.6084/m9.figshare.12998549.v3

***Methods:***

***The inclusion of older adults with Dementia (n=3), Cognitive Decline (n=3), Parkinson's Disease (n=1), and Cognitive frailty (n=1) can be a possible limitation of the present study, considering the differences related to comorbidity. I suggest the author exclude these studies. Neuropsychiatric symptoms in MCI and Dementia are related to several different mechanisms, as well as the psychological and physiological effects of physical exercise in these populations. The characteristics of Depression in these cases can be very specific, with causes, neurophysiological changes and treatments that differ from depressive symptoms in healthy older adults. The inclusion of these studies can contribute of shrinking the effect size of exercise. For example, the effectiveness of pharmacological approaches to managing depressive symptoms in Dementia and MCI is less effective and the antidepressant effect of exercise in these populations is still controversial.***

 We agree with the Reviewers comments relating to the antidepressant effects of exercise, and in particular that evidence in these populations remains controversial. Before undertaking this review, specific inclusion criteria for this work were considered greatly, wherein, the authors remained agnostic to any particular effect size resulting from exercise participation on depressive symptoms in older adults. The purpose of the present review was to provide point-specific information that is generalisable to all older adults aged >65 years without diagnosis of clinical depression.

 The binary approach taken within this review was (a) without diagnosis of clinical depression, thus excluding (b) those diagnosed with clinical depression. This review included RCTs limited to the former, which became the defining characteristic. Specifically, we cannot bias the inclusion criteria based on ubiquitous conditions, and as such, any given adult aged >65 years is by definition a primary candidate for each of the suggested limiting covariates.

 We are genuinely grateful to this Reviewer for generating some important points relating to exercise gerontology which remain unresolved. Until such time as epidemiological controversy such as nuanced and unresolved characteristics amongst ageing cohorts is evidenced, it is our belief that we must accept perceived imperfections within the literature, which is a notable limitation of meta-analytical investigation. This Reviewer does imply an important fact, and we do not offer this article as being definitive as such potential limitations are unresolved. However, we do believe that it is best practice to present in the article’s current form, but importantly, accept and acknowledge the value of the Reviewer’s observation by noting this as a generalised limitation within the limitations section. By doing this, there is opportunity for researchers to improve on this research, should new evidence add confirmatory knowledge. Supplementary sensitivity analysis specific to this can be found in the online repository, located here:

- ‘Data Availability’ > ‘Extended data’ > ‘Additional Sensitivity Analysis’
- https://doi.org/10.6084/m9.figshare.12998549.v3

***If the authors decide to include these population (MCI, Parkinson, Dementia), several other studies that investigated the effect of exercise on neuropsychiatric symptoms (such as depression) in patients with MCI and Dementia should be included in the present meta-analysis, For more details, see Liang 2018 and Leng et al., 2018. ^1^ Moreover, this issue should be included in the limitation section.***

 We are further grateful to this Reviewer for providing a list of potential references and have applied article inclusion criteria in review of each. From this list, one study (i.e., Abd El-Kader & Al-Jiffri, 2016) met the inclusion criteria for the systematic review but was not eligible for inclusion in network meta-analytics. Thus, the Systematic Review component of this article has been updated, yet the network meta-analysis remains unchanged. In response to the Reviewer’s recommendation, we have carefully examined the review conducted by Leng et al. (2018). We have applied the present article inclusion criteria in review of each study and further included detailed reasoning for inclusion/exclusion for each which may be in the online repository, each of which may be observed at:

 ‘Data Availability’ > ‘Extended data’ > ‘Suggested Articles Provided During Peer Review’

- https://doi.org/10.6084/m9.figshare.12998549.v3

***Factors related to the prescription of exercise (frequency, intensity, duration, length, supervision, cognitive engagement) should be more explored in analyses and results.***

 This is an important point to consider (and partially addressed in the previous comment). One primary difference between network meta-analysis and its more widely used counterpart, the ‘meta-analysis’, relates to the limited ability for network meta-analysis to address significant covariates by sub-group analytics and meta-regression of known moderators of the primary outcome. However, such important factors have a place to address this within network meta-analysis. Although not overtly observable, we have done so within the present article. More specifically, “in order to test transitivity across networks, potential effect modifiers were tested with meta-regression sensitivity analyses for the entire pool of studies and separately for each exercise comparison (aerobic vs. resistance vs. mind-body). No significant modifying effects were observed for the pool of studies or any separate exercise comparison for age, gender, source of participants, length of intervention, format of program, exercise intensity, frequency, duration, adherence, year of study, risk of bias, publication status, intention-to-treat analysis, nor cluster design, indicating that the assumption of transitivity was upheld.”

 Further information can also be found in the online repository, found here:

- ‘Data Availability’ > ‘Extended data’ > ‘Supplementary File S1’
- https://doi.org/10.6084/m9.figshare.12998549.v3

***Discussion:***

***The author should discuss the possible different mechanisms related to the antidepressant effect of mind-body practices, aerobic and resistance training in older adults, considering both psychological and physiological hypotheses. Please consider more recent neurobiological mechanisms related to the effect of exercise on depressive symptoms (e.g., myokines, neurotrophins, inflammatory cytokines, etc.).***

 Possible different mechanisms, and unique characteristics relating to the antidepressant potential of different forms of exercise, remain to be fully defined. The main purpose of meta-analytical review of randomised controlled trials (RCTs) is to leverage the work of other researchers, and by doing so improve statistical power and point estimation for a specific question. In this respect, the present review cannot offer psychological or physiological hypotheses to a degree greater than any one specific RCT.

 However, we do accept this Reviewer’s guidance and further accept that it is important to contextualise contemporary mechanisms relating to the antidepressant effect of exercise. In light of this, we have included a separate extended piece of 784 words within the online repository to provide an extension of this discussion, found here:

- ‘Data Availability’ > ‘Extended data’ > ‘Supplementary File S1’
- https://doi.org/10.6084/m9.figshare.12998549.v3

Additionally, the authors have added the following text “we direct the reader to the Extended data for further discussion relating to potential mechanisms for the antidepressant effects of exercise in older adults”, to add value to this overall body of work and contemporary mechanistic information for the interested reader.

***Conclusion:***

***The authors concluded: “Pooled RCT evidence highlights that each individual exercise mode (aerobic, resistance, and mind-body) can effectively mitigate symptoms of depression in older adults, irrespective of depression severity”. I suggest excluding the phrase “irrespective of depression severity”, since the authors did not run a specific analysis to evaluate this issue.***

 Thank you for drawing our attention to this critical oversight from the authors. The authors agree and this has now been removed for reasons of specific accuracy. Specifically, this has been moderated and subsequently amended within the Abstract, Discussion, and Conclusion sections.

References

 Abd El-Kader SM, Al-Jiffri OH: Aerobic exercise improves quality of life, psychological well-being and systemic inflammation in subjects with Alzheimer’s disease. Afri Health Sci. 2016;16(4): 1045-55. 28479898 10.4314/ahs.v16i4.22

 Leng MA, Liang B, Zhou H, et al.: Effects of physical exercise on depressive symptoms in patients with cognitive impairment: A systematic review and meta-analysis. J Nerv Ment Dis. 2018;206: 809–23. 30273278 10.1097/NMD.0000000000000887

# Peer review: Reviewer response for version 1

**DOI:** 10.5256/f1000research.29961.r80099

**Published:** 2021-03-08

**Authors:** Walid Kamal Abdelbasset

**Affiliations:** 1 Department of Health and Rehabilitation Sciences, College of Applied Medical Sciences, Prince Sattam bin Abdulaziz University, Al-Kharj, Saudi Arabia; 2 Department of Physical Therapy, Kasr Al-Aini Hospital, Cairo University, Cairo, Egypt

This study aims to examine the head-to-head effectiveness of aerobic, resistance, and mind-body exercise to mitigate depressive symptoms in adults aged ≥ 65 years. The topic is interesting, however, some corrections should be addressed.

**General comments:**

- The English presentation needs to be improved throughout the manuscript.
- This study is a systematic review. Therefore, transfer kindly the PRISMA checklist, which can help clarify the missed information.

**Specific comments:**

 Introduction:

- Define depression in detail. Determine the relationship between exercise and depression status in older adults.  I suggest the authors expose the following points in the introduction: What is known about depression and exercise training in older adults? What is not known? Why the study was done? The hypothesis is also missed in this section.

Discussion:

- The main findings should be presented in the introductory paragraph with respect to the study objectives and hypothesis.
-  Inconsistency models need more details in the discussion section.
- Please, re-frame the discussion along the following lines:
  Main findings of the present study
  Comparison with other studies
  Implication and explanation of findings
  Strengths and limitations
  Conclusion, recommendation, and future direction.

Are the rationale for, and objectives of, the Systematic Review clearly stated?

Partly

Is the statistical analysis and its interpretation appropriate?

Yes

Are sufficient details of the methods and analysis provided to allow replication by others?

Yes

Are the conclusions drawn adequately supported by the results presented in the review?

Partly

Reviewer Expertise:

Exercise Intervention; Geriatric Rehabilitation; Rehabilitation Sciences

I confirm that I have read this submission and believe that I have an appropriate level of expertise to confirm that it is of an acceptable scientific standard, however I have significant reservations, as outlined above.

# Response

**Published:** 2021-06-30

**Authors:** Kyle Miller

**Affiliations:** Federation University, Australia

***This study aims to examine the head-to-head effectiveness of aerobic, resistance, and mind-body exercise to mitigate depressive symptoms in adults aged ≥ 65 years. The topic is interesting, however, some corrections should be addressed.***

 We extend our gratitude to the Reviewer for their critique and offering an opportunity to improve our article. The author team have taken the time to reflect on each comment and reached consensus, which are provided in detail below.

***General comments:***

- ***The English presentation needs to be improved throughout the manuscript.***

The majority of the authorship team are native English language speakers and while each of us acknowledge that we are in continual development, we are experienced in communicating scientific writing in our native language. However, we do acknowledge this criticism in the spirt of improvement that it is meant. Thus, we have taken time to consider literary communication and addressed to the best of our collective ability.

  

- ***This study is a systematic review. Therefore, transfer kindly the PRISMA checklist, which can help clarify the missed information.***

Should we be accurate in the interpretation of this comment, the Reviewer considers this article to be (i) a systematic review. This is correct. However, it is a systematic review combined with (ii) network meta-analysis, which requires compliance with a specific set of reporting guidelines that are an extension of the noted PRISMA checklist. This article is presented in acknowledgement of a complimentary extension to PRISMA guidelines where we have adhered to the ‘PRISMA-NMA guidelines’, where ‘NMA’ accounts for a quantitative addition within this ‘network meta-analysis’. To support our response of confirmatory compliance, we direct the Reviewer to the Methodology section within this article which specifies a formal statement of compliance with PRISMA-NMA guidelines. To further offer confirmatory compliance with PRISMA-NMA guidelines, we direct this Reviewer to supplementary materials within the online repository, found here:

- ‘Data Availability’ à ‘Reporting Guidelines’ à ‘PRISMA-NMA Checklist’
- https://doi.org/10.6084/m9.figshare.12998549.v3

***Specific comments:***

***Introduction:***

***Define depression in detail.***

 We thank the Reviewer for identifying potentially important rationale for conducting this systematic review and network meta-analysis. In order to provide a comprehensive response to the query, it is necessary to offer two components of ‘depression’:

- Definition of depression – This is an interesting request, and the authors believe it to be adequately rationalised within the Introduction section. In compliance PRISMA-NMA criteria, we have specified the generalised term ‘depression’ as having a characteristic binary format for this work where explicitly (i) ‘clinically defined depression’ is divergent with (ii) ‘depressive symptoms’ which fall short of the threshold for the former. The present article focuses on the latter, where we offer contextual dialogue of literature within the domain of ‘clinical depression’ and articulate within the Discussion section of this article.
- Operational definition for this article – The discrete definition for depressive symptoms used in this review are further detailed within the methodology, where RCTs were deemed eligible where valid determination of depressive symptoms was achieved using accepted psychometrically valid tools.

***Determine the relationship between exercise and depression status in older adults.  I suggest the authors expose the following points in the introduction:***

- ***What is known about depression and exercise training in older adults? What is not known?***

We direct the Reviewer to Paragraphs 2 of the Introduction section: “International public health consortia are in concert with the antidepressant effects of exercise as a low-risk adjunct for optimal mental health (American Psychiatric Association, 2010; Stubbs et al., 2018; World Health Organization, 2010). While we do not yet have the answers for low uptake of exercise in older adults (Knowles et al., 2015), it may in some way be due to nuanced regimen design, which in turn, may similarly impact compliance in exercise prescription by primary and stakeholders in aged care.”

 Similarly, Paragraph 3 of the same Introduction section: “During the past four decades, widely different metabolic, social, and environmental demands between exercise modalities (i.e., running vs. weightlifting vs. Tai Chi) have been well-characterised. Given that there is variation between exercise regimens, and these variations are not merely semantics, one may be surprised to discover that only a few randomised controlled trials (RCTs) have deliberately compared the antidepressant effects of different exercise regimens in older adults (Martins et al., 2011; Penninx et al., 2002).”

 The authors acknowledge that may not be ideal for the Reviewer and are optimistic that the body of referenced material in coalition with the short steps of logical reasoning, offer mitigatory satisfaction of this Reviewer’s exposed points.

  

- ***Why the study was done?***

We agree that this is an important attribute which must be present within empirically sound research efforts. Accordingly, we direct the Reviewer to view the final paragraph within the Introduction section, which reads: “The purpose of this systematic review and network meta-analysis was to quantitively assess the best evidence from RCTs to establish relative (head-to-head) effectiveness of resistance, aerobic, and mind-body exercise in adults aged ≥ 65 years, and below the clinical threshold for diagnosed depression. More specifically, we investigated whether (i) resistance, aerobic, and mind-body exercise training can induce substantive treatment effect on depressive symptoms in older adults, (ii) while considering relative treatment compliance to each exercise regimen, and further, (iii) to juxtapose the optimal exercise treatment for all adults aged ≥ 65 years irrespective of depressive symptomology.”

  

- ***The hypothesis is also missed in this section.***

We are thankful to the Reviewer for addressing this important consideration. PRIMA-NMA guidelines stipulate that those wishing to conduct meta-analytical review should “provide an explicit statement of questions being addressed, with reference to participants, interventions, comparisons, outcomes, and study design (PICOS).”

 There is opportunity to investigate this further, where we direct the Reviewer the emergent work from Bruce Charlton titled ‘The used and abuses of meta-analysis’, wherein it is prescribed that “meta-analysis is not a hypothesis-testing activity” and where Charlton further offers that meta-analysis “cannot legitimately be used to establish the reality of a putative hazard or therapy” (Charlton, 1996). In this respect, we prefer to present the research questions in its current active format.

 More generally, we offer that the majority of the author team are experimental scientists where research is beset on the presentation of formal hypotheses. We agree that it could be a route to detail a specific question of focus. However, as (for example) the present work relies upon and leverages the efforts from many RCT authors to satisfy our independent question, we must also acknowledge that their individual nuanced differences become amplified to induce collective heterogeneity towards reaching a resolution to our research question. For reasons such as this, we selected to side with formal guidelines (in this case, PRISMA-NMA) to achieve the outcomes detailed within the present article.

***Discussion:***

- ***The main findings should be presented in the introductory paragraph with respect to the study objectives and hypothesis.***

The authors offer some satisfaction to resolving the ‘hypothesis’ component of the Reviewer’s suggestion in the prior response. In terms of addressing the substantive part of this Reviewer’s query, we offer the main findings from this review to be presented at the beginning of the Discussion section, and specifically, in the inaugural paragraph, which reads: “The present review offers new information for general exercise prescription to support mental health outcomes for adults aged ≥ 65 years. Specifically, (i) aerobic, resistance, and mind-body each demonstrate equivalent benefit to mitigate symptoms of depression in adults aged ≥ 65 years, (ii) compliance to exercise treatment is notably encouraging for each exercise types, and (iii) when combined with the pool of data from clinically depressed older adults the effectiveness of aerobic, resistance, and mind-body exercise is comparably consistent for all adults aged ≥ 65 years, irrespective of depression severity. These findings should provide reassurance for personnel and stakeholders in healthy ageing to encourage exercise prescription from a point of pragmatism and in collaboration with patient preference.”

  

- ***Inconsistency models need more details in the discussion section.***

Within the present article, inconsistency models are detailed within the Results section (see ‘Assessment of Inconsistency’). Our generalised ambition in delivering coherence within the Discussion section was to integrate our findings from inconsistency modelling and to communicate in a more active voice in appreciative context with other available literature. As it turned out, our tests of inconsistency became largely satisfied with generalised assumption of consistency for (i) global and (ii) loop-specific heterogeneity for our main (depressive symptom) network which limited an expanded discussion of inconsistency. To support this Reviewer’s query and in our compliance with Cochrane Review guidelines (Higgins et al., 2021), network inconsistency was further interrogated using the GRADE assessment tool (Salanti et al., 2014). In doing this, any important inconsistencies are dialogued in context to the primary objective, which in turn, are dialogued within the Discussion section and benchmarked against our primary outcomes. For instance, Paragraph 5 of the Discussion section reads: “Certainty of evidence is moderate, due to the dispersion in effect size estimates resulting in imprecision.”

***Please, re-frame the discussion along the following lines:***

- ***Main findings of the present study***
- ***Comparison with other studies***
- ***Implication and explanation of findings***
- ***Strengths and limitations***
- ***Conclusion, recommendation, and future direction.***

The authors have partially satisfied this request (RE: ‘Specific Comments - Discussion’), with requisite response above. In compliance with previously identified guidelines, we have attempted to provide a coherent Discussion section in this article which is presented in the following generalised flow:

- Paragraph 1: Summary of main findings.
- Paragraph 2-6: Theoretical implications and comparisons with past studies.
- Paragraph 7-10: Practical considerations and recommendations.
- Paragraph 11-13: Strengths, limitations, and future directions.
- Paragraph 14: Conclusion and final recommendation.

We thank this Reviewer for constructive critique and the authors are satisfied that we have an improved article with support from Reviewer commentary.

References

 American Psychiatric Association: Practice guideline for the treatment of patients with Major Depressive Disorder. 3 ed. Washington, DC: American Psychiatric Association;2010.

 Charlton BG: The uses and abuses of meta-analysis. Family Practice. 1996;13(4): 397–401. 8872100 10.1093/fampra/13.4.397

 Higgins JPT, Thomas J, Chandler J, et al.: Cochrane handbook for systematic reviews of interventions version 6.2 (updated February 2021). Cochrane, 2021. Available from www.training.cochrane.org/handbook

 Knowles AM, Herbert P, Easton C, et al.: Impact of low-volume, high-intensity interval training on maximal aerobic capacity, health-related quality of life and motivation to exercise in ageing men. Age (Dordr). 2015;37(2):25. 25773069 10.1007/s11357-015-9763-3 4359174

 Martins R, Silva MCE, Pindus D, et al.: Effects of strength and aerobic-based training on functional fitness, mood and the relationship between fatness and mood in older adults. J Sports Med Phys Fitness. 2011;51(3):489–96. 21904289

 Penninx BWJH, Rejeski WJ, Pandya J, et al.: Exercise and depressive symptoms: A comparison of aerobic and resistance exercise effects on emotional and physical function in older persons with high and low depressive symptomatology. J Gerontol B Psychol Sci Soc Sci. 2002;57(2):P124–32. 11867660 10.1093/geronb/57.2.p124

 Salanti G, Del Giovane C, Chaimani A, et al.: Evaluating the quality of evidence from a network meta-analysis. PLoS One. 2014;9(7):e99682. 24992266 10.1371/journal.pone.0099682 4084629

 Stubbs B, Vancampfort D, Hallgren M, et al.: EPA guidance on physical activity as a treatment for severe mental illness: A meta-review of the evidence and Position Statement from the European Psychiatric Association (EPA), supported by the International Organization of Physical Therapists in Mental Health (IOPTMH). Eur Psychiatry. 2018;54:124–44. 30257806 10.1016/j.eurpsy.2018.07.004

 World Health Organization: Global recommendations on physical activity for health. Geneva, Switzerland: World Health Organization;2010.
